# A multilocus phylogeny reveals deep lineages within African galagids (Primates: Galagidae)

**DOI:** 10.1186/1471-2148-14-72

**Published:** 2014-04-02

**Authors:** Luca Pozzi, Todd R Disotell, Judith C Masters

**Affiliations:** 1 Department of Anthropology, Center for the Study of Human Origins, New York University, New York, New York, USA; 2 New York Consortium in Evolutionary Primatology, New York, USA; 3 Behavioral Ecology and Sociobiology Unit, German Primate Center, Göttingen, Germany; 4 African Primate Initiative for Ecology and Speciation, Department of Zoology and Entomology, University of Fort Hare, Alice, South Africa

**Keywords:** Concatenation, Species tree, Divergence times, Nuclear DNA, Eocene-Oligocene boundary, Strepsirhini, Lorisoidea

## Abstract

**Background:**

Bushbabies (Galagidae) are among the most morphologically cryptic of all primates and their diversity and relationships are some of the most longstanding problems in primatology. Our knowledge of galagid evolutionary history has been limited by a lack of appropriate molecular data and a paucity of fossils. Most phylogenetic studies have produced conflicting results for many clades, and even the relationships among genera remain uncertain. To clarify galagid evolutionary history, we assembled the largest molecular dataset for galagos to date by sequencing 27 independent loci. We inferred phylogenetic relationships using concatenated maximum-likelihood and Bayesian analyses, and also coalescent-based species tree methods to account for gene tree heterogeneity due to incomplete lineage sorting.

**Results:**

The genus *Euoticus* was identified as sister taxon to the rest of the galagids and the genus *Galagoides* was not recovered as monophyletic, suggesting that a new generic name for the Zanzibar complex is required. Despite the amount of genetic data collected in this study, the monophyly of the family Lorisidae remained poorly supported, probably due to the short internode between the Lorisidae/Galagidae split and the origin of the African and Asian lorisid clades. One major result was the relatively old origin for the most recent common ancestor of all living galagids soon after the Eocene-Oligocene boundary.

**Conclusions:**

Using a multilocus approach, our results suggest an early origin for the crown Galagidae, soon after the Eocene-Oligocene boundary, making *Euoticus* one of the oldest lineages within extant Primates. This result also implies that one – or possibly more – stem radiations diverged in the Late Eocene and persisted for several million years alongside members of the crown group.

## Background

African galagids (Family Galagidae) are small, nocturnal primates widely distributed in sub-Saharan Africa, from as far west as Senegal (*Galago senegalensis*) to Somalia in the east (*Galago gallarum*), and from as far north as southern Sudan (*Galago senegalensis*) to South Africa (*Otolemur crassicaudatus*). Members of the family Galagidae, commonly known as galagos or bushbabies, show a diverse set of adaptations in their diet, ecology, and social behavior [1,2]. Their body masses range from that of the Rondo galago (*Galagoides rondoensis*), one of the smallest living primates (~60 g), to the cat-sized greater galago (*Otolemur crassicaudatus*) weighing up to 2 kg [1-3]. With such a wide range of body mass, galagos show a high diversity of dietary adaptations, including feeding on insects (up to 70% for the smallest species), flowers, fruits, exudates, and gum [1,2]. For instance, the medium-sized needle-clawed galagos (*Euoticus* spp.) base up to 75% of their diet on gum [1,2,4]. In general, the social systems of galagos have been poorly studied. Originally thought to be solitary, nocturnal strepsirhines are now viewed as having social structures based on dispersed “social networks” revealed by sleeping associations, most often involving females. Within this framework, authors have described social organizations that combine solitary foraging with one male-multifemale sleeping associations [4], dispersed multi-male social structures (e.g. *Otolemur* spp. [5,6]), where males have larger territories and related females cluster together in small groups, and dispersed monogamy (e.g. *Galagoides cocos*[6,7]), whereby one male/one female or one male/two or three females form associations [1,2,5-7].

Because of their nocturnal habits and often inaccessible locations, galagos are one of the most understudied groups of primates and little is known about the biology of most species. Species diversity has long been underestimated because of a lack of overt morphological diversity [1,8]. Over the last two decades, several new morphologically cryptic species have been reported based primarily on advertisement calls. Vocal signals used in mate attraction are likely to be reliable indicators of species identity and have been used extensively in taxonomic studies of primates, including gibbons [9-11], tamarins [12,13], tarsiers [14], guenons [15,16], and colobines [17,18]. Traditionally, only five species and two genera of galagos were recognized, *Euoticus* and *Galago*[19]. More recently, at least three additional genera (*Otolemur*, *Galagoides,* and *Sciurocheirus*) and almost twenty new species have been described [1,2,20].

**Figure 1:**
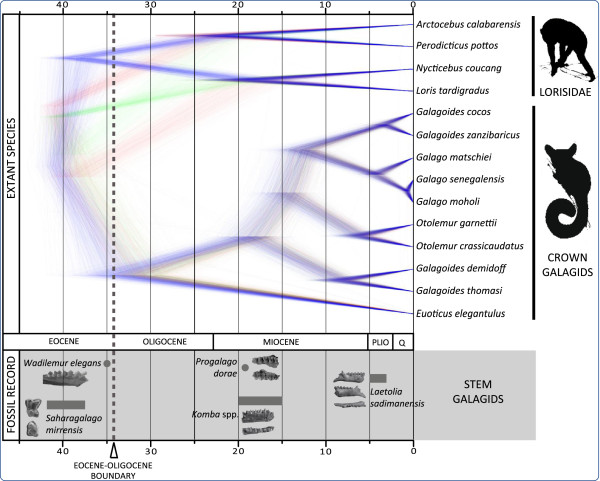
**Previous phylogenetic hypotheses of galagid relationships.** Grey boxes indicate the position of members of the genus *Galagoides* while arrows indicate the position of *Euoticus*. **(a)** Galagid phylogeny based on 40 characters including morphology, reproductive and vocal behavior from Groves (2001) [23]. **(b)** Phylogeny based on a supermatrix of mitochondrial and nuclear DNA from Fabre et al. (2009) [27]. **(c)** Phylogenetic reconstruction based on a concatenation of nuclear gene segments and mitochondrial gene sequences from Springer et al. (2012) [28]. *The authors did not recognize the genus *Galagoides*, which is subsumed within the genus *Galago*.

The increase in named species within galagids has affected dwarf galagos in particular. Nash et al. [21] and Kingdon [3] included all dwarf galagos within the genus *Galagoides*: *i.e.* small forest species with body mass < 200 g, and with shorter hindlimbs than members of the genus *Galago*. They differ, too, in several skull characteristics not found in *Galago*[22]. While *Galagoides demidoff* and *Galagoides thomasi* inhabit central and western Africa, several of the dwarf galago species recognized more recently are restricted to East Africa [3]. At least six different species have been described in this region: *Galagoides cocos* along the coastal forest of Kenya and Somalia; *Galagoides granti* from Tanzania to Mozambique in the south; *Galagoides nyasae* inland near Lake Malawi; *Galagoides zanzibaricus udzungwensis* in central and coastal Tanzania and *G. z. zanzibaricus* on the island of Zanzibar; *Galagoides orinus* in many of the Eastern Arc Mountains of Kenya and Tanzania; and *Galagoides rondoensis* in a few isolated patches of coastal forest in Tanzania. However, the validity of the genus *Galagoides* is still uncertain and several morphological [23] and molecular studies [24-28] have failed to support its monophyly (Figure 1). Groves [23] preferred to merge all ‘*Galagoides*’ species into the genus *Galago* (Figure 1a)*,* while Masters et al. [26] found the genus to be paraphyletic and suggested that a new generic designation for the Zanzibar group (*Galagoides zanzibaricus-cocos-granti*) would be required.

Various studies have attempted to clarify the phylogenetic relationships within galagids by using morphological or molecular data, or a combination of the two [23-31] (Figure 1). However, a clear picture of galagid phylogeny has been elusive and the relationships among genera are still debated. Besides the taxonomic validity of *Galagoides*, another major source of disagreement is the position of the enigmatic needle-clawed galago (*Euoticus* spp.) (Figure 1). Some molecular studies based on mitochondrial DNA found *Euoticus* closely related to members of *Galago*[31], and more specifically, the sister species of *Galago matschiei*[25,27] (Figure 1b). Masters et al. [26] used a combination of molecular and morphological characters and placed the genus *Euoticus* as sister taxon to *Galago*, a position also supported by Groves [23] on the basis of morphological and behavioral traits (although he submerged the genus *Galagoides* within the genus *Galago*) (Figure 1b)*.* An alternative view of galagid phylogeny was proposed by Stiner and Turmelle [30]. In their analysis of partial mitochondrial DNA sequences (cytochrome *b*, 12S and 16S rRNAs), they reconstructed *Euoticus* as the basal divergence with no particular relationship to *Galago*. This hypothesis was supported in a more comprehensive molecular study of primates conducted by Springer et al. [28] (Figure 1c). The basal position of needle-clawed galagos raises interesting questions about the adaptations and evolutionary history of the entire family*.* As stated above, Springer et al. [28] also failed to infer *Galagoides* monophyly, with the eastern species (represented in their study by *Galagoides orinus*, *Galagoides zanzibaricus*, and *Galagoides granti*) clustering together with *Otolemur* + *Sciurocheirus.* Despite their inclusion of multiple genes and species, the dataset of Springer et al. [28] had a lot of missing data (several species were represented by only one or a few loci) and the support for many nodes within Galagidae was extremely low (bootstrap values < 50%).

Another important open question about the evolutionary history of galagids is the time of their divergence. The paleontological record for crown galagids is quite sparse and mainly restricted to a few Pliocene-Pleistocene species in eastern Africa, such as *Otolemur howelli* (Shungura formation, Omo, Ethiopia, ~3.0-3.2 Ma [32]) and possibly some specimens belonging to *Galago senegalensis* (Olduvai Gorge, Tanzania, ~1.8 Ma [33]) and *Galagoides cf. zanzibaricus* (Omo, Ethiopia, ~3.0 Ma [32]). A possible exception is *Galago farafraensis* found in Sheikh Abdallah, Egypt and dated as Late Miocene (~10-11 Ma) [34]. This species is known from several isolated teeth and postcranial elements which are similar in morphology to *Galago senegalensis*, but more like *Galagoides demidoff* in size. Another Miocene galagid, represented by a single mandible, was found in the Tugen Hills (Lukeino formation) in Kenya, and is dated around 6 Ma [35,36]. However, the phylogenetic placement of this fossil specimen is still uncertain [37]. Finally, “*Galago*” *sadimanensis*, once considered part of the crown radiation, is now placed in its own genus, *Laetolia* (Laetoli, ~3.5-5.0 Ma) and probably represents a primitive sister taxon to crown galagids [38].

While no crown galagids are known from sediments older than the Late Miocene/Early Pliocene, the oldest occurrence of stem members of this family date back to the Late Eocene, when it is represented by two species found in the sediments at Fayum, Egypt: *Saharagalago mirrensis* (Fayum, ~36.9-42 Ma [39]) and *Wadilemur elegans* (Fayum, ~35 Ma [40]). The putative lorisid *Karanisia clarki* from the later Eocene, initially interpreted as closely related to the genus *Arctocebus*, is now considered a stem lorisiform [39,40]. The occurrence of *Saharagalago mirrensis* at ~37 Ma suggests that lorises and galagos had diverged by the close of the Middle Eocene [41,42]. Other stem galagids from East Africa, including members of the genera *Progalago* (~19 Ma [43,44]) and *Komba* (15–20 Ma [36,44,45]), are dated more recently, as Early-Middle Miocene [37]. The phylogenetic placement of *Progalago*, however, is still debated, with authors classifying it as a stem galagid [37,38] or as a crown lorisiform of uncertain affinities [46,47].

Many recent molecular studies have used the stem galagid *Saharagalago* to date the divergence between Lorisidae and Galagidae, and have suggested Late Oligocene/Early Miocene origins for crown galagids. Fabre et al. [27] estimated the origin of crown galagids at ~25 Ma, while Springer et al. [28] placed it at ~23 Ma. Molecular studies also suggest fairly deep divergences among the main lineages within the family. For instance, the genus *Otolemur* was estimated to have diverged approximately 8 Ma, while the common ancestor of the members of *Galagoides* in eastern Africa (*zanzibaricus-granti-orinus*) was estimated to have lived between 7.5-10 Ma *e.g.*[27,28].

To clarify phylogenetic relationships within the family Galagidae, and specifically the position of *Euoticus* and the taxonomic validity of the genus *Galagoides*, we obtained DNA sequence data representing the main lineages within Galagidae for 27 independent nuclear loci. As an initial step we performed maximum-likelihood and Bayesian concatenated phylogenetic analyses, and used Bayesian relaxed-clock methods to infer dates for the diversification of the galagid family. The concatenation of multiple loci has been used extensively in primatology [27,28,31,48,49] although, despite the practical advantages of this approach, both simulation and empirical studies have shown that this method can perform poorly in cases of high tree discordance across different loci [50-52]. More specifically, phylogenetic reconstructions based on concatenated datasets do not account for individual gene histories, and can therefore produce misleading topologies, with most of the nodes highly supported (high bootstrap values and/or posterior probabilities) despite their not reflecting the actual evolutionary history of the species [52-56]; but see also [57].

Alternatively, gene tree-species tree methods, which use a coalescence approach, take into account the possible discordance among genes – mainly as a consequence of incomplete lineage sorting (ILS) – and reconstruct the species tree within which each individual gene tree is embedded [52,58,59]. Coalescence methods have recently been applied to primate phylogenies [60-64], and are likely to provide a more realistic picture of the primate tree [62,65]. Hence, as a second step, we applied a coalescence-based species tree approach to phylogenetic inference within galagids, and compared our results to those obtained from concatenated analyses.

## Results

### Concatenated analyses

**Figure 2:**
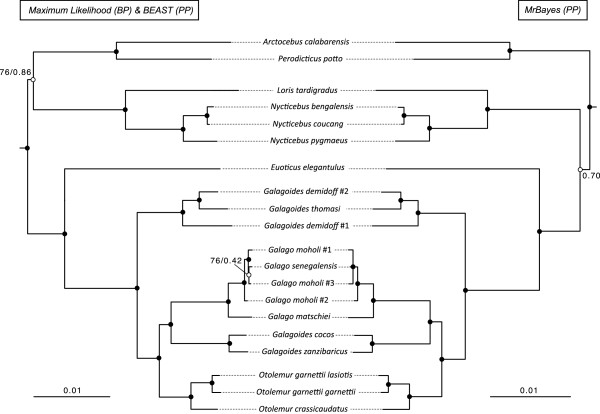
**Phylogenetic trees inferred from the concatenated dataset based on maximum likelihood (RAxML) and BEAST on the left and MrBayes on the right.** Black circles indicate nodes that were strongly supported in all analyses (BP ≥70% and PP ≥ 0.95), while white circles indicate nodes in which support was low (BP < 70% and/or PP < 0.95). Specific values for those nodes that were poorly supported in the analyses are reported on the trees.

Maximum likelihood (ML) and Bayesian (MB and BEAST) analyses yielded slightly different topology estimates (Figure 2 and Additional file 1). While the monophyletic status of the family Galagidae was maximally supported by all the analyses (bootstrap probability (BP) = 100% and posterior probability (PP) = 1.00), the family Lorisidae was inferred as monophyletic only in ML and BEAST analyses, but with relatively low support (BP = 76% and PP = 0.86, respectively). In contrast, an alternative topology with the Asian lorisids (*Loris* and *Nycticebus*) more closely related to galagids than to the African lorisids (*Perodicticus* and *Arctocebus*) was recovered in Bayesian analyses, also with low support (PP = 0.70).

Branch lengths across the tree were comparable between different analyses (RAxML, MrBayes, and BEAST) and showed a short internode between the Lorisidae/Galagidae split and the origin of the African and the Asian lorisid clades (Figure 2 and Additional file 1). Within the Lorisidae, both the Asian (*Nycticebus* + *Loris*) and the African (*Arctocebus* + *Perodicticus*) clades were inferred as monophyletic with high support across all analyses (BP = 100% and PP = 1.00).

Within galagids, all analyses found maximal support for most of the nodes. The genus *Euoticus* was strongly supported as the sister taxon of all other galagids (basal divergence within the family; BP = 100% and PP = 1.00), rather than being closely related to the genus *Galago*, as suggested in some previous studies. All analyses found the genus *Galagoides* not to be monophyletic. Both maximum likelihood and Bayesian analyses supported two distinct clades within “*Galagoides*”: one included the species *Galagoides demidoff* and *Galagoides thomasi* (hereafter referred as the western clade) (BP = 100% and PP = 1.00) and the other included *Galagoides cocos* and *Galagoides zanzibaricus* (the eastern clade; BP = 100% and PP = 1.00). The western clade was maximally supported (BP = 100% and PP = 1.00) as the sister taxon to a clade including *Otolemur*, *Galago* and the *Galagoides* eastern clade. Within this clade, members of the eastern clade were strongly supported as the sister group of the genus *Galago* (including *Galago senegalensis, Galago moholi*, and *Galago matschiei*) (BP = 96% and PP = 1.00), to the exclusion of members of the genus *Otolemur* (*O. crassicaudatus* + *O. garnettii*) (BP = 100% and PP = 1.00).

### Coalescence-based species tree analyses

To evaluate support across individual loci for the nodes inferred by the concatenated analyses, we ran MrBayes analyses for each locus. Strong support or topological congruence between individual loci and the concatenated tree is not necessarily expected since the level of congruence may be affected by several factors, including homoplasy, low levels of variation, or gene tree heterogeneity. However, even moderate support across many loci can provide evidence that concatenated results are not driven by only a few genes. In order to evaluate the effect of missing data in the gene tree-species analyses, we compiled two dataset with 27 (hereafter 27LOCI) and 19 loci (hereafter 19LOCI), respectively. The latter dataset was reduced to only 19 loci in order to avoid missing data at the locus level (every taxon was represented for all 19 loci; see Methods for details).

Bayesian analyses of individual genes generally supported some clades, including the monophyly of Lorisoidea, African lorisids, Asian lorisids, and some sister relationships at the species level (*Galagoides thomasi* and *Galagoides demidoff*, and *Galagoides cocos* and *Galagoides zanzibaricus*) in both datasets (27LOCI and 19LOCI). Several loci, however, yielded poor resolution and low support for most nodes (see Additional file 2). For instance, the loci APP and ZIC3 showed high support (PP > 0.95) for only two and three nodes out of 13, respectively. Average levels of support (*i.e.* PP) across loci ranged from 0.21 to 1.00 in the dataset 27LOCI, and between 0.26 and 0.99 in the dataset 19LOCI. The average posterior probability across loci was higher than 0.70 for four out of 13 nodes in the dataset 27LOCI (~31%) and for seven out of 12 nodes in the dataset set 19LOCI (58%). Except for the root (Node 1), the highest level of support across loci was found for the sister taxon relationship between *Galagoides cocos* and *Galagoides zanzibaricus*, with an average PP of 0.91 (27LOCI: 77.8% loci with PP > 0.95) and 0.94 (19LOCI: ~95% loci with PP > 0.95). In both datasets, the two nodes that showed the lowest support in the individual gene analyses were Node 2 (relationships among African lorisids, Asian lorisids, and galagids) and Node 9 (relationships among *Galago*, *Otolemur*, and the eastern *Galagoides* clade), with average PP ranging between 0.05 and 0.29 (see Additional file 2). For these two nodes, several loci supported alternative topologies. For instance, in the 27LOCI dataset, eight loci supported the relationship between Asian lorisids and galagids (node 2A: PP = 0.20 [0.45-0.99]), eleven loci supported the monophyly of lorisids (node 2C: PP = 0.24 [0.34-0.87]), and only two loci supported the sister relationship between African lorisids and galagids (node 2B: PP = 0.05 [0.26-1.00]) (see Additional file 2). A similar result was inferred for the dataset 19LOCI, with average support of 0.26 for Node 2A (7 loci [0.36-0.93]), 0.06 for Node 2B (3 loci [0.22-0.70]), and 0.20 for node 2C (6 loci [0.34-0.84]).

**Figure 3:**
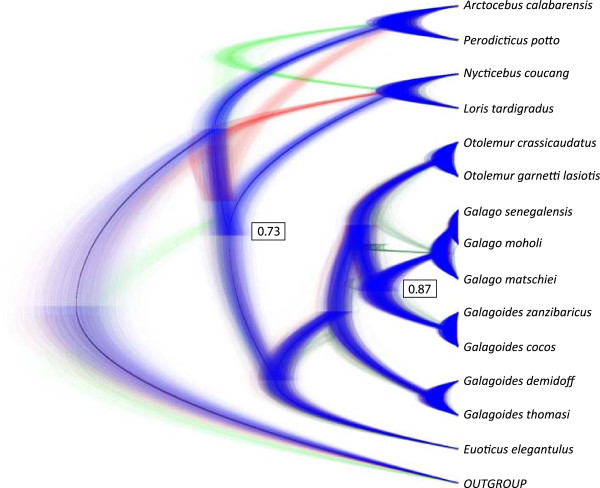
**Densitree **[66]**showing the posterior probability of 75000 trees from coalescent-based species tree analyses on 27 loci using BEST.** Blue represents the trees sharing the most probable topology (70.97% of trees), red represents the second most probable topology (19.15% of trees) and green represents the third most probable topology (9.85% of trees). Bayesian posterior probability was greater than 0.95 for all nodes, except for the two nodes indicated in the figure.

Overall, gene tree-species tree analyses yielded similar results to the concatenated analyses. The BEST (Bayesian Estimation of Species Trees) analyses did not support the monophyly of the family Lorisidae, but a sister relationship between Asian lorisids and galagids, to the exclusion of African lorisids. However, support for this node was relatively low in both datasets analyzed (27LOCI: PP = 0.73; 19LOCI: PP = 0.65) (Figure 3 and Additional files 1 and 3). Within Galagidae, all gene tree-species tree methods inferred the same topology as concatenated analyses. Most of the nodes within Galagidae were inferred with maximal support (PP = 1.00) in both datasets. The only node with relatively lower support in the gene tree-species tree analyses was the sister relationship between the genus *Galago* and the clade including *Galagoides cocos* and *Galagoides zanzibaricus* (27LOCI: PP = 0.87 and 19LOCI: PP = 0.87) (Additional files 1 and 3).

### Divergence time estimates

**Figure 4:**
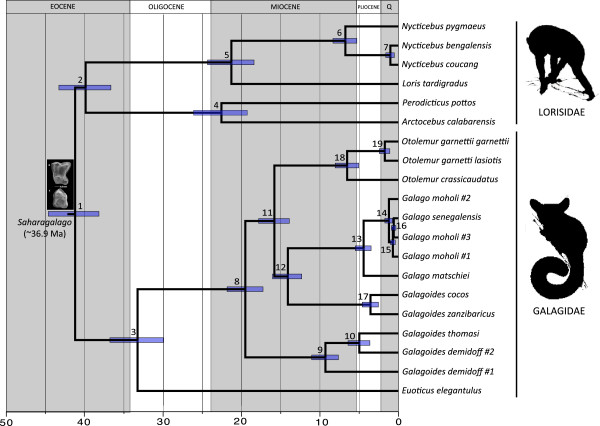
**Single chronogram with divergence date estimates from 27 concatenated loci.** For each node confidence bars indicating the 95% highest probability densities (HPDs) are reported (see Additional file 1 for the actual dates and 95% HPDs for all nodes).

Dating analyses were run on the concatenated dataset. Visual inspection of parameter estimates from BEAST runs with and without data, respectively, showed markedly different values. This suggests that the data, and not the initial priors alone, are informing the results. BEAST analyses estimated the origin of crown Lorisoidea at 41.25 Ma (95% HPD = 38.22-44.68) and the origin of Lorisidae to be around 39.92 Ma (95% HPD = 36.70-43.33). The origin of crown galagids, represented in this study by the split between *Euoticus* and the remaining galagid species, was estimated to be Early Oligocene, approximately 33 Ma (33.29 Ma; 95% HPD = 29.96-36.82) (Figure 4 and Additional file 1). Interestingly, all the other lineages within the family Galagidae fall into a single clade with the most recent common ancestor estimated at ~19 Ma, during the Early Miocene (19.54 Ma; 95% HPD = 17.29-21.87), roughly 14 Ma after the origin of the crown group. The splits between *Otolemur* and *Galago* + *Galagoides* (eastern clade) and *Galago* and *Galagoides* (eastern clade) are estimated to have occurred in the Middle Miocene, approximately 15 Ma (15.84 Ma; 95% HPD = 13.93-17.85) and 14 Ma (14.12 Ma; 95% HPD = 12.34-16.09), respectively (Figure 4 and Additional file 1).

Divergence estimates between sister species were unexpectedly old for most of the taxa analyzed in this dataset*: Galagoides cocos* and *Galagoides zanzibaricus* diverged ~3.5 Ma (3.58 Ma; 95% HPD = 2.57-4.63) and *O. garnettii* and *O. crassicaudatus* at ~6.5 Ma (6.56 Ma; 95% HPD = 5.06-8.09). In contrast, the clade containing *Galago moholi* and *Galago senegalensis* was estimated to be quite recent (1.23 Ma; 0.77-1.75); however, *Galago moholi* was inferred to be paraphyletic in both ML and MB analyses conducted on this dataset (Figure 2). *Galagoides demidoff* was also inferred to be paraphyletic in the analyses, and the divergence among the three specimens in the western clade (two *Galagoides demidoff* and one *thomasi*) was dated as Late Miocene, approximately 9 Ma (9.33 Ma; 95% HPD = 7.67-11.09). Estimates for all nodes in the BEAST tree are presented in Additional file 1, along with 95% HPD intervals.

## Discussion

In this study, we have provided new molecular data to assess phylogenetic relationships and divergence dates within the family Galagidae. We assembled the largest molecular dataset for galagos to date by sequencing 27 independent loci totaling > 18,000 base pairs. With the exception of the genus *Sciurocheirus*, all known major galagid lineages were included in the study.

### Phylogenetic conclusions

Our phylogenetic analyses showed strong support for most of the nodes across both Galagidae and Lorisidae. However, weak support was found for the relationships between galagids, and African and Asian lorisids. The family Lorisidae was inferred to be either monophyletic (ML and BEAST) or paraphyletic, with a sister relationship between Asian lorises and galagos (MB), although both arrangements were relatively poorly supported across all analyses. Similarly, the gene tree-species tree analyses inferred a sister relationship between Asian lorisids and galagids, to the exclusion of the African lorisids, with low support. The analyses of individual loci showed a high level of discordance across different genes, and, in most cases, the loci that supported one or other hypothesis showed low posterior probability values.

The interrelationships among members of Lorisoidea have been problematic and little agreement has been reached across studies [67]. Morphologically, lorises represent probably one of “the best-diagnosed clades within primates” [68]. Members of this group share a large number of putative morphological synapomorphies of the skull (e.g. raised temporal lines), dentition (e.g. diminution of M^3^), and postcrania (e.g. reduction of the tail; fore- and hindlimbs of near equal length; *retia mirabilia* in wrists and ankles) [69-73]. In addition to morphological traits, numerous behavioral, physiological, and ecological characteristics link Asian and African lorises [4,46,71-73]. Several of these shared traits are related to "slow-climbing" locomotion [23]. However, the validity of the lorisid clade has been challenged by several molecular studies which failed to support the monophyletic status of the family, suggesting a close relationship between galagids and either Asian ([25] (cytochrome *b*), [26,31,68,74-76]; this study) or African lorisids [77,78]. The only unambiguous molecular evidence to date that supports lorisid monophyly is the shared presence of three mobile elements (SINEs [25]). Although SINEs have been proposed to be good phylogenetic markers [79-81], in the presence of short internodes they can be affected by ILS [80,82]. While most molecular studies have found lorises to be paraphyletic, the resolution of this node has been neither consistent nor robust [25,27,28,31,68,75,76,79]. More recently, two studies involving multiple loci combined into a single matrix supported the monophyly of the family Lorisidae [28,49], but only in Perelman et al. [49] this topology was strongly supported.

The phylogenetic analyses conducted in this study show a relatively short internode between the origins of the crown lorisoids and the divergence of the two families, Lorisidae and Galagidae. The short internal branch that separates the crown lorisoids and the crown lorisids has been pointed out by several authors [25,47,68,75], and it may be one of the reasons why molecular data have failed to provide convincing support for any of the three alternative topologies. Despite the inclusion of a large number of loci and a comprehensive taxonomic sampling of lorisoids, our study also failed to resolve the relationships among Asian lorises, African lorises, and galagids with any reliability. Gene tree-species tree analyses provided some support for the sister relationship between Asian lorises and galagos, but more data are needed to clarify this issue. Future phylogenomic studies should include longer loci in order to test the hypothesis of ILS in a coalescence framework. Long – and likely more informative - loci have been suggested as advantageous for species tree estimation, especially when coalescent methods are used [56].

The short length of the branch dividing crown lorisoids from lorisids raises interesting questions about the evolutionary history of this group. If lorises are indeed monophyletic, all their shared morphological, physiological, and ecological adaptations must have evolved relatively rapidly. Alternatively, the "slow-climbing" features of lorisid anatomy may be plesiomorphic for all lorisoids [74]. Both the fossil record and reconstructions of the ancestral morphotype support the idea of a more generalized ancestor, with a progressive morphological separation between two related clades, the ‘slow-climbing’ lorises and the ‘fast-leaping’ galagos [46,47]. A third hypothesis is that the unique morphological features shared by the African and Asian lorisids evolved in parallel in the two clades [83], making the lorises one of the most remarkable cases of parallel evolution within primates [47,68].

Our phylogenetic analyses help to clarify several aspects of galagid relationships. First, this study provides strong support for the basal position of the enigmatic genus *Euoticus* within Galagidae [28,30] as opposed to a sister relationship between the needle-clawed galagos and members of the genus *Galago*[25-27,31]. A possible explanation for these two alternative reconstructions is that ILS may affect the phylogenetic placement of *Euoticus*. All the studies that supported the close relationship between *Euoticus* and *Galago* were based on mitochondrial DNA, and it is possible that mitochondrial phylogeny disagrees with the rest of the genome, as is known for other primate groups [76,78]. Unfortunately, complete mitochondrial genomes are available for only two galagid genera (*Otolemur* and *Galago*), and further studies are needed to explore the level of incongruence between mitochondrial and nuclear data. However, we believe ILS to be unlikely in this specific instance. First, the study conducted by Stiner and Turmelle [30] was also based on mitochondrial sequences and, since the mitochondrial genome behaves like a single locus, it is difficult to explain the discrepancy between phylogenies based on the same marker. Second, ILS is more likely to occur in cases of short internodes (little time between speciation events) and/or high effective population sizes [50-52]. The branch lengths inferred from this study showed very long internodes between *Euoticus* and members of the genus *Galago.* An alternate hypothesis is that the inaccurate taxonomic allocation of *Euoticus* specimens may have affected some phylogenetic reconstructions. Inaccurate identifications of specimens in museum collections are quite common within galagids [8, Masters and Couette, pers. comm.].

A close relationship between needle-clawed galagos and lesser galagos has also been suggested by some morphological studies [71,72]. Members of *Euoticus* and *Galago* share some similarities in their cheek tooth and skull morphology, including a marked degree of basicranial flexion and a short, square snout [72]. Based on these morphological similarities and the sister-group relationship with members of the genus *Galago*, Masters et al. [26] suggested downgrading *Euoticus* to a subgenus of *Galago*. However, *Euoticus* also shows some traits in common with lorises, probably as an adaptation to strengthen the skull morphology for bark chewing and gum scraping [72]. These potential convergences make the phylogenetic placement of *Euoticus* uncertain based solely on morphological traits. For instance, Masters and Brothers [72] found the position of *Euoticus* to switch from basal in the galagid tree to the sister-taxon of *Galago* as a consequence of outgroup choice or weighting scheme for morphological characters. Our study strongly supports the basal divergence of *Euoticus* within the family, which provides a possible explanation for the fact that *Euoticus* has anomalously short tarsal regions, particularly when compared with *Galago*, which contains the most specialized leapers in the family [8]. According to our reconstruction, *Euoticus* is likely to have diverged before the major tarsal elongation took place in Galagidae. Future studies will include more specimens of this genus to confirm its phylogenetic placement within the galagids, and also to investigate the validity of its two putative species, *E. pallidus* and *E. elegantulus*.

Our results also strongly indicate that the genus *Galagoides* is not monophyletic*.* Members of this genus belong to two independent clades, one including *Galagoides demidoff*-*Galagoides thomasi* (western clade) and the other including *Galagoides zanzibaricus-Galagoides cocos* (eastern clade)*.* The western clade was recovered as the sister group of all the other galagids except *Euoticus*, while the eastern clade was strongly supported as the sister taxon of the genus *Galago.*

Analyses of morphological data have traditionally supported the monophyly of this genus (e.g., [26,72,84]), but most analyses of molecular data have contradicted this finding ([24-28,31], this study). As a consequence, *Galagoides* has been reported as a “wastebasket taxon of plesiomorphic species” [24,26,30]. Our study further supports the hypothesis that *Galagoides* is not monophyletic and the genus *Galagoides*, *sensu stricto* (including only *demidoff* and *thomasi*), represents an independent clade from both *Galago* and the remaining ‘*Galagoides’* (eastern clade).

The generic name *Galagoides* was first used to describe *Galagoides demidoff*[85], but no name is available for members of the *zanzibaricus* group. The sister group relationship between *Galago* and ‘*Galagoides’* (eastern clade) suggests the possibility of including the Zanzibar galagos within the genus *Galago*. Although previous studies classified *Galagoides zanzibaricus* as a subspecies of *Galago senegalensis*, it is clearly not only a distinct species [21,23,86], but deserves to be separated at a generic level. The definition of a genus is somewhat arbitrary but most authorities agree that a genus should be monophyletic and occupy “an ecological situation - or adaptive zone - that is different from that occupied by the species of another genus” [87]. Although they form a monophyletic group, Zanzibar galagos differ from the lesser galagos (*Galago*) in several aspects: smaller body size (usually < 200 g), shorter limbs and lighter build, and characters of the skull and teeth [22]. Species of the genus *Galago* are usually restricted to dry woodlands and savannahs (with the exception of *Galago matschiei*), while the Zanzibar galagos inhabit the lowland and coastal forests of eastern Africa. Acoustically *Galago* spp. do not give buzz calls, and their recognition calls are highly variable in length [88]. We posit that morphological, behavioral, and ecological differences indicate that the Zanzibar galagos should be placed in a new genus for which a new name is required (Masters et al., *in preparation*). The two species *thomasi* and *demidoff* would remain in the genus *Galagoides*, while the new genus would include all members of the Zanzibar complex (*zanzibaricus*, *cocos*, and *granti*). ‘*Galagoides’ granti* was not included in this study but all studies conducted to date have strongly supported its close affiliation with ‘*Galagoides*’ *zanzibaricus*[27-29,31]. No clear classification is available for the other species currently ascribed to the genus *Galagoides* due to the absence of genetic data. To date only ‘*Galagoides*’ *orinus* has been included in phylogenetic studies, and it was inferred as the sister taxon to the Zanzibar clade [28,29]. If this topology is confirmed, ‘*Galagoides*’ *orinus* should be reclassified within the new genus; however, a new designation would be premature at this stage, since those phylogenetic reconstructions were based on a limited amount of genetic data (partial 12S rRNA). No genetic data are currently available for ‘*Galagoides*’ *rondoensis*, ‘*Galagoides*’ *nyasae,* and the still undescribed Mt. Rungwe galago (‘*Galagoides*’ sp. nov.) [20]. Future studies should include these species of dwarf galagos in order to test their phylogenetic placement in relation to the Zanzibar group.

The Zanzibar dwarf galagos (‘*Galagoides*’) were strongly supported as sister taxon of *Galago* (lesser galagos), to the exclusion of *Otolemur* (greater galagos). This result agrees with other molecular [25,27,31] and morphological studies [23,72]. However, the interrelationships among the clades ‘*Galagoides*’, *Galago*, and *Otolemur* have been challenging to resolve, and several studies have suggested two alternative topologies: *Otolemur* is either the sister taxon of *Galago*[24,26] or the sister taxon of the Zanzibar galagos [28,89]. Although all the analyses in this study produced the same topology for the three genera, with ‘*Galagoides*’ more closely related to *Galago* than to *Otolemur*, we offer a caveat. Analyses based on concatenated datasets supported this relationship very strongly, while gene tree-species tree methods revealed a high amount of gene tree discordance for this node. This suggests the confidence placed in this node by concatenated analyses may have been overestimated. Alternative topologies with *Otolemur* as the sister taxon of either *Galago* or ‘*Galagoides*’ received some support from multiple loci, indicating a level of gene heterogeneity. Once again, the branch lengths that separate the three clades are relatively short and it is possible that ILS is masking some of the phylogenetic signal at these nodes. However, as indicated by the analyses conducted for individual loci, it is also possible that other factors, such as the different substitution rates and low level of phylogenetic information in several loci, may affect our ability to resolve this node when individual loci are analyzed separately or in a coalescence framework (see also [57]).

### Divergence dates

Estimated divergence times for the origins of crown Galagidae have varied in recent molecular studies. Previous studies suggested Late Oligocene-Early Miocene origins for the crown group, ranging between 20 and 26 Ma [27,28,31,49]. Roos et al. [25] estimated the age of crown galagids slightly older, around ~30 Ma, calibrated using their estimate of 61 Ma (50–80 Ma) for the split between Lorisiformes and the Malagasy lemurs. However, this date is not based on fossil evidence.

Our age estimates are somewhat older for crown Galagidae, and indicate the group originated just after the Eocene-Oligocene boundary (EOB). Based on the nuclear data analyzed in this paper, *Euoticus* represents an ancient lineage estimated around 33 Ma old (Figure 4 and Additional file 1), approximately 14 Ma prior to the origin of rest of the crown group (~19.5 Ma), in the Early Miocene. This estimate is only slightly younger than the dates for the crown Galagidae obtained by other studies in which *Euoticus* was not basal in the tree or was not included in the study [27,31,49]. In those phylogenetic reconstructions, the origin of the crown group was represented by the emergence of the clade including *Galagoides thomasi/demidoff*, and accords well with our date for this divergence. *Euoticus* is thus a critical taxon for understanding the evolutionary history of galagos; the phylogenetic position of *Euoticus* within galagids can be considered analogous to that of *Daubentonia* within lemurs (e.g. [28,49]): both taxa represent ancient lineages that diverged a considerable period (> 10 Ma) before the rest of the crown group radiation.

The origin of crown galagids just after the EOB raises interesting questions about the evolution of this group, and more generally, that of all African Strepsirhini. The beginning of the Oligocene (around 33.9 Ma) coincides with a climatic change from the relatively warm and wet conditions of the Eocene to the cooler, drier conditions in the Oligocene [41,90]. Although the levels of extinction at the EOB were not as catastrophic as previous events (e.g. Cretaceous–Paleogene mass extinction), the fossil record documents a gradual decrease in primate diversity throughout the Late Eocene and the Early Oligocene [41]. While this period was characterized by long-term cooling at high latitudes in Europe and North America (also known as the “Grande Coupure” in Europe), the EOB is associated with a major floristic change in equatorial Africa [91] and an increased aridity in the north [41,92]. This climatic change is correlated with the disappearance of at least four strepsirhine clades (including Galagidae) from the Fayum sediments of Egypt [41]. Galagos clearly persisted across the EOB, but no fossils between 35–37 and the mid-Miocene (~10 Ma) have been found in northern Africa [34,41]. Although the absence of evidence does not necessarily imply true extinctions, it seems clear that the strepsirhine community underwent a dramatic restructuring in the Oligocene, as shown by the Early Miocene record [41]. The dates obtained in this study suggest that crown galagos originated soon after the EOB (~33 Ma) with the divergence of the lineage leading to *Euoticus*. Given the presence of two West African lineages at the base of the tree (*Euoticus* and the *Galagoides thomasi/demidoff* clade), it is possible that the origin of the crown Galagidae occurred in central-western Africa, where equatorial rain forests were still likely to be widespread during the Early Oligocene. Central-western African origins for crown galagids might also explain the absence of galagids in the fossil record between ~35 Ma (*Wadilemur*) and ~20 Ma (*Komba* and *Progalago*). The fossil record for primates in western Africa is notoriously poor because forested habitats do not provide ideal conditions for fossilization.

Another possible indication of West African origins for the crown galagids relates to the number of lineages surviving in that region: except for the eastern clade of the genus ‘*Galagoides’,* possibly restricted to eastern Africa, most genera are either present (*Galago* and *Galagoides*) or restricted (*Sciurocheirus* and *Euoticus*) to central-western Africa. The high species diversity in eastern Africa, especially within the dwarf galagos, is likely to be more recent and related to climatic and ecological changes during the Late Miocene and Early Pliocene in the Eastern Arc Mountains and coastal forests [93,94].

An interesting aspect of the date estimates we obtained is the lack of divergences in the crown group between the Early Oligocene (the divergence of *Euoticus*) and the Early Miocene (the split between *Galagoides* spp. and the lineage leading to *Otolemur*, *‘Galagoides’* and *Galago*). The presence of stem galagids at around 15–20 Ma (*Komba* and *Progalago*) implies that these lineages survived independently for 20–30 Ma through the later Eocene and Oligocene into the Early Miocene (and possibly even mid-Pliocene if *Laetolia sadimanensis* is indeed a stem galagid [38]), while members of the crown group were completely unsampled until at least ~10 Ma (*Galago farafraensis*). The systematics of the Early Miocene East African lorisoids *Komba* and *Progalago* has long been debated, and studies have reached different conclusions, including the taxa as stem or crown members of Galagidae and Lorisidae, or advanced stem or very basal crown lorisiforms [46,95,96]. Most recent studies seem to support *Komba* as a stem galagid [37-40] but the taxonomic status of *Progalago* has remained ambiguous, identified either as a stem galagid [37,38] or a crown lorisiform of uncertain affinities [46,47]. Unfortunately, *Euoticus* was not included in several of these phylogenetic studies and the relationships between this taxon and the putative stem galagids is still ambiguous. If *Komba*, and possibly *Progalago* and *Laetolia,* are correctly classified as stem galagids, at least one stem radiation (but possibly more) took place before the EOB, and some members persisted for several million years beside crown members.

Finally, the divergence estimates for some of the sister species in this study were relatively old. The two species of *Otolemur*, *O. crassicaudatus* and *O. garnettii*, apparently diverged in the Late Miocene, approximately 6.5 Ma. This estimate agrees with some previous studies that support an old origin for this split [27,31,49]. Several studies that included a third species of *Otolemur*, *O. monteiri*, push the origins of the genus back to ~10 Ma [27,28], although the validity of *O. monteiri* is still unclear, and further studies on the systematics of *Otolemur* using molecular data are required (see [23]). The divergence between the Zanzibar galagos (*‘Galagoides*’ *zanzibaricus*) and the Kenya Coast galagos (*‘Galagoides*’ *cocos*) is estimated to be approximately 3.5 Ma*. ‘Galagoides*’ *cocos* has recently been elevated to full species status based on acoustic data [20,97], although its taxonomic validity is still uncertain. This old divergence is interesting considering that the taxa are morphologically very similar, offering support for the hypothesis that speciation in galagos is driven by changes in specific-mate recognition signals, particularly vocalizations [88,89,97-100].

In contrast, the clade including *Galago senegalensis* and *Galago moholi* was inferred to be quite recent (~1.2 Ma), as suggested by Masters [101]. These woodland species occupy different areas of floral endemism, and it is possible that speciation in lesser galagos might have taken place alongside that of their plant hosts, in response to the increasing aridity during the Middle Pleistocene [101]. Our study recovered *Galago moholi* as paraphyletic, possibly as a consequence of taxonomic misclassification of GenBank sequences and/or captive animals. Similarly to museum specimens, samples from captive sources are often incorrectly classified and this problem is particularly relevant for lesser galagos, the taxa most commonly found in captivity. More genetic and biogeographic studies, possibly with samples collected directly from wild populations, are therefore needed to elucidate patterns of speciation in lesser galagos.

## Conclusion

Galagids are one of the least studied groups of primates and little is known about their evolutionary history and phylogeny. This lack of knowledge is primarily due to the limited genetic data available for most species. Here, we present a new molecular study of African galagos based on 27 independent loci, and present a generally well-supported phylogeny for this group. At the phylogenetic level, our two main results are (1) the basal position of *Euoticus* in the galagid tree; and (2) the non-monophyletic status of *Galagoides*. As a consequence, we suggest that a new generic designation for the Zanzibar group (here represented by the two species *zanzibaricus* and *cocos*) is required. Also, given its phylogenetic position, *Euoticus* represents a taxon of critical importance to studies of the evolutionary history of galagos. Despite the amount of genetic data collected for this study, the monophyly of the family Lorisidae remained unsupported and requires further investigation. Our results suggest an early origin for the crown Galagidae, soon after the Eocene-Oligocene boundary, implying that one – or possibly more – stem radiations, including fossils like *Komba*, *Progalago*, and *Laetolia*, diverged in the Late Eocene and persisted for several million years alongside members of the crown group. Based on the age estimates obtained in this study, *Euoticus* represents one of the oldest lineages within Primates, and its divergence during the Early Oligocene appears to be independent of the radiation that gave rise to all the other main galagid lineages later in the Miocene.

## Methods

Twenty taxa were sampled within Lorisoidea, along with ten primate outgroup species. The ingroup included six lorisids (6 species – 4 genera) and 14 galagids (10 species – 4 genera). DNA sequence data were obtained from a total of 27 independent nuclear loci, ranging from 351 bp to 1295 bp (Table 1). These loci were selected from Perelman et al. [49] based on the performance of the primers across all the samples. A list of the primers used for each locus is presented in Additional file 4. Some sequences were used in previous phylogenetic studies of primates [49], but 233 new sequences were generated for this study, and assembled together with 264 sequences for five species of galagids and six of lorisids obtained from Perelman et al. [49]. All new sequences were deposited in GenBank under accession numbers presented in Additional file 5. Some samples could not be amplified for some loci; nevertheless, within lorisoids, taxon coverage for individual genes varied from 70% to 100% (average 92%; Table 1) and the final dataset included 12.1% missing data. Ten primate taxa, three lemurs (*Daubentonia madagascariensis*, *Lemur catta*, and *Propithecus verreauxi*) and seven catarrhines (*Homo sapiens, Pan troglodytes, Pongo pygmaeus, Macaca mulatta, Papio hamadryas, Theropithecus gelada,* and *Chlorocebus aethiops)* were selected as outgroup taxa. The final dataset included 30 taxa (species and subspecies) representing most of the major lineages within Lorisoidea (eight genera out of nine). A list of the samples used in this study is provided in Additional file 6.

**Table 1:** List of loci used in this study with characteristics and taxon coverage (number of species sampled)

**Locus**	**Length (bp)**	**Taxon coverage**		**Lorisoidea**		**Galagidae**		**Model**
		**Lorisoidea (20)**	**Galagidae (14)**	**VS**	**PIS**	**VS**	**PIS**	
ABCA1	674	14	9	104	52	58	22	GTR + I
ADORA3	416	18	12	42	28	17	10	HKY + G
AFF2	510	19	13	43	28	27	17	GTR + I
APP	714	17	13	40	20	20	8	GTR + G
ATXN7	565	19	14	72	47	42	21	SYM + G
AXIN1	951	19	13	67	33	32	12	GTR + I + G
BCOR	789	19	13	69	51	48	34	HKY + I + G
CHRNA1	425	19	13	79	41	38	18	HKY + G
DACH1	630	19	13	114	47	59	18	GTR + G
DCTN2	635	19	13	76	46	47	19	K80 + G
DENND5A	747	20	14	159	80	82	41	HKY + G
ERC2	793	17	11	139	86	60	33	GTR + G
FAM123B	747	19	14	181	79	81	41	SYM + G
FBN1	735	20	14	53	29	23	7	HKY + I
GHR	1295	14	10	164	90	96	33	HKY + G
KCNMA1	656	16	10	46	31	26	15	GTR + I
LRPPRC-171	819	18	13	103	52	45	25	HKY + G
LUC7L	751	19	13	77	42	36	14	HKY + G
NPAS3.2	680	20	14	121	64	73	22	GTR + G
PNOC	351	18	13	57	29	41	15	HKY + G
POLA1	658	20	14	71	37	47	21	GTR + I
RAG2	769	19	13	73	42	50	24	HKY + G
RPGRIP1	713	18	12	85	38	49	11	HKY + G
SGMS1	616	20	14	34	13	17	5	GTR + I
SIM1	670	20	14	42	18	26	12	HKY + I
SMCX	365	18	14	56	21	42	12	HKY + G
ZIC3	574	19	13	40	14	18	2	HKY + G
TOTAL	18248	18.4 (92.0%)	12.8 (91.5%)	2119	1150	1157	511	

Note: VS: Variable Sites.

PIS: Parsimony-Informative Sites.

### Ethical Statement

Most samples were not specifically acquired for this study. Samples were provided by the American Museum of Natural History in New York City and the Duke University Lemur Center, or were obtained from wild animals, and had been used in earlier molecular studies [24,26]. Only samples from *Otolemur garnettii lasiotis*, ‘*Galagoides*’ *cocos*, and ‘*Galagoides*’ *zanzibaricus* were obtained from wild animals specifically for this study. Wild samples were collected between 2010 and 2012 from two different sites in Kenya (Diani Forest, −4°19', +39°34') and Tanzania (Udzungwa National Park, −7°52', +36°51'). The animals were captured using Tomahawk live traps baited with fruit, insect larvae, and palm wine (e.g. [4,5,7,100]). Up to 20 traps were set at dusk between ground level and 5 m., and checked 4–5 times during the night. To limit stress, individual animals were handled for a maximum of 20 minutes. Hair samples and approximately 2 mm^2^ ear biopsies were taken from each individual and preserved in sterilized 2 ml tubes filled with RNAlater buffer. All animals were released at the exact site of capture immediately after sample collection. Permission for fieldwork and sample collection was provided by the Ministry of Education, Science and Technology in Kenya and the Tanzania Wildlife Research Institute (TAWIRI) in Tanzania to LP. CITES export permits were obtained from both Kenya and Tanzania. Sample collection was approved by the University Animal Welfare Committee (UAWC) at NYU (IACUC animal care protocol #10-1334) and adhered to the American Society of Primatologists (ASP) Principles for the Ethical Treatment of Non-Human Primates (see https://www.asp.org/society/resolutions/EthicalTreatmentOfNonHumanPrimates.cfm). No animals were sacrificed for this study.

### DNA isolation and sequencing

DNA was extracted and isolated from tissue samples (either ear clips from live animals, or a small snip of muscle from dead animals) using the QIAamp DNA Micro Kit (Qiagen, Inc.) following the protocol provided by the manufacturer. For some samples only a limited amount of DNA was available. In these cases, whole genome applications (WGA) were used for the downstream analyses. WGAs were performed using REPLI-g Mini Kits (Qiagen). Between 50–100 ng of genomic DNA were used for each 50 ml reaction following the manufacturer’s protocol. A negative control was included in every WGA and was verified by downstream PCR and sequencing.

PCR amplification of all nuclear gene regions was carried out using either AmpliTaq Gold® 360 Master Mix (ABI) or AccuPrime™ *Taq* DNA Polymerase System (Invitrogen™). For the first kit, PCRs were performed in a reaction volume of 15 μL and a reaction mix consisting of 7.5 μL of AmpliTaq Gold® 360 Master Mix, 5.4 μL of water, and 0.3 μL (10 μM) of each primer. For the AccuPrime reactions, the mix consisted of 2.0 μL of 10× Buffer II, 0.08 μL of AccuPrime™ *Taq* (5 U/μL)*,* and 0.4 μL (10 μM) of each primer.

PCR reactions were carried out using a touchdown program with the following parameters: 95°C for 2 min, followed by a first round of 25 cycles denaturing at 95°C for 15 s, primer annealing starting at 60°C (and gradually decreasing to 50°C over 25 cycles) for 30 s, and extension at 72°C for 1 min; and followed by a final round of 25 cycles of 95°C for 15 s, 50°C for 30s, and 72°C for 1 min; and a final extension at 72°C for 7 min. The initial denaturation was extended to 10 min for the AmpliTaq Gold® 360 Master Mix protocol.

PCR products were analyzed on 1% agarose gels. PCR products that produced clear single bands were purified using ExoSAP-IT for PCR Product Clean-Up (Affymetrix) and then sequenced directly in two reactions with forward and reverse primers (the same as the amplification primers). The sequencing reactions were carried out with the BigDye Terminator v3.1 cycle sequencing kit (Applied Biosystems, Inc). The cycle sequencing reactions were performed in a reaction volume of 10 μL and a reaction mix consisting of 1.5 μL of 5X Sequencing buffer, 0.5-0.7 μL of BigDye, 1.2 μL (10 μM) of each primer and 1.0 μL of PCR product. Sequencing reactions were performed with 50 cycles at 96°C for 10 s, 50°C for 5 s, 60°C for 4 min. Finally, sequencing products were analyzed on an ABI 3730 DNA Analyzer system (Applied Biosystems, Inc.) and bases were called using Sequencing Analysis v5.2 (Applied Biosystems, Inc.). Consensus sequences for each individual were generated from sequences in forward and reverse directions using Geneious R6.1 (Biomatters).

### Sequence alignment

Each locus was first aligned independently using MUSCLE [102], and then combined in a single matrix resulting in a total alignment length of 18,248 base pairs (bp). A second alignment was performed to remove poorly aligned regions in the dataset using Gblocks 0.91b [103] under a relaxed approach. Poorly aligned regions can interfere with phylogenetic reconstructions by adding noise to the analyses, and their removal can improve the performance of phylogenetic reconstructions, especially in studies including very divergent sequences [103,104]. Gblocks was run with the options “Minimum Length Of A Block” = 10 and “Allowed Gap Positions” = “With Half”. The final alignment after running Gblocks consisted in 14,372 bp (78% of the original alignment). Both alignments (full and Gblocks) are available on TreeBase (http://purl.org/phylo/treebase/phylows/study/TB2:S15281).

### Phylogenetic analysis

Phylogenetic analyses were conducted on the partitioned concatenated dataset under maximum likelihood (ML) and Bayesian inference (MB). ML analyses were run using a separate partition for each locus (27 partitions in total). We used Randomized Accelerated Maximum Likelihood in RAxML version 7.2.6 [105,106]. For each partition scheme, we ran 50 independent ML inferences (using 50 distinct randomized MP trees) with a GTR + G model to estimate the best topology. In order to assess the support for individual branches we performed both a rapid (−*f a -x* option) and non-parametric bootstrap (−*b* option) with 1000 replications to assess support on different nodes [106,107]. Maximum-likelihood bootstrap proportions (BP) ≥70% were considered strong support [108,109].

Bayesian analyses were performed using MrBayes 3.2.2 [110] with the Metropolis coupled Markov Chain Monte Carlo (MCMC) algorithm. The best-fitting model of nucleotide evolution was selected independently for each partition using the Akaike Information Criterion (AIC) as implemented in MrModelTest 2.3 [111] as reported in Table 1. Posterior probability (PP) support values higher than 0.95 were considered strong support for individual clades [112-114]. Four separate MrBayes runs, each including four incrementally heated chains, were run for 20 million generations. Within each run, convergence was assessed by checking LnL, the average standard deviation of split frequencies (< 0.01), and the potential scale reduction factor (PSRF) in MrBayes. We also assessed convergence visually using Tracer v.1.5 [115] to plot the likelihood versus generation number and estimate the effective sample size (ESS > 200) of all parameters, and to compare the performance of the four independent analyses. Finally, we used AWTY [116] to plot pairwise split frequencies for the four independent MCMC runs and to check the posterior probabilities of clades for non-overlapping samples of trees in the sample using the compare and slide commands, respectively. After checking for convergence, we summarized the posterior distribution of trees, removing the first 25% of generations as burn-in. All RAxML and MrBayes analyses were performed via the High Performance Computing (HPC) clusters at New York University.

### Species tree analyses

Coalescence-based species tree analyses were performed using BEST (Bayesian Estimation of Species Trees) v2.3 [117]. This software uses MrBayes [110,118] to estimate separate gene trees while simultaneously estimating the species tree that generated them. This method accounts for uncertainty in the individually estimated gene trees, and it also allows the separate gene tree estimates to influence each other during the analysis [117].

BEST analyses were run on the same dataset of 27 nuclear loci described above (27LOCI), although the dataset was reduced from 30 to 16 taxa: one member for each species was selected within Galagidae (10 taxa) and one member per genus within Lorisidae (four taxa), plus two lemurs, *Lemur catta* and *Propithecus verreauxi*, as outgroups. We restricted the analysis to 16 taxa because gene tree-species tree analyses are computationally intensive and a larger dataset could have made it difficult or impossible to reach proper convergence among repeated analyses [60,61,119]. Taxon coverage for individual genes varied from 81.3% (13 out of 16 taxa for ABCA1) to 100% (average 94.7%) (see Additional file 7: Table S7a).

Gene tree-species tree methods use information for each individual locus to estimate the species tree; therefore, it is important to minimize the amount of missing data in the dataset. Missing data may interfere with the proper estimation of individual gene trees and affect the inference of the most likely species tree for that set of loci [119,120]. In order to explore the effect of missing loci/taxa, we compiled a second dataset, which included 15 taxa and 19 loci (19LOCI) (see Additional file 7: Table S7b). All taxa were represented for each of the 19 loci and missing data were only present within each individual locus (differences in sequence length). This second dataset included all major lineages within lorisids (4 taxa) and galagids (9 taxa), plus two outgroups (*Lemur catta* and *Propithecus verreauxi*).

BEST analyses were performed by setting α = 3 and β = 0.003 of the inverse gamma distribution prior on effective population size (h). We ran four separate analyses with different random starting points and two chains per run (one cold and one heated), and compared the results across runs [119]. We ran the analyses for 100 million generations for the dataset 19LOCI (sampling every 1000 generations) and 150 million generations for the dataset 27LOCI (sampling every 1000 generations). After checking for convergence across independent runs, the species trees files (*.sptree*) were combined and summarized, excluding the first 25% of generations as burn-in.

### Divergence-time analyses

We performed dating estimates using the uncorrelated Bayesian relaxed-clock method as implemented in BEAST v1.7.5 [121,122]. BEAST simultaneously estimates the tree topology and divergence times. As in the previous analyses using MrBayes, posterior probability (PP) values greater than 0.95 were considered strong support. BEAUTi v1.7.5 (part of the BEAST package) was used to prepare the *.xml* file for use with BEAST v1.7.5 [121,122]. Evolutionary rates along branches followed an uncorrelated lognormal distribution, and a birth-death speciation process was used for all analyses [123]. Four replicate runs were conducted with four MCMC chains sampled every 1000 generations for 60 million generations, after a burn-in period of 15 million generations (equivalent to 25%). Convergence was checked using Tracer v1.5 and all BEAST analyses were run to achieve an effective sample size (ESS) of at least 200 for all estimated parameters once burn-in was removed. Results from the four independent runs were then combined using LogCombiner, and maximum credibility trees with divergence time means and 95% highest probability densities (HPDs) were produced using TreeAnnotator v1.7.5 [121]. In order to check the influence of the priors on the results, analyses were all run without data and were compared to those with data using Tracer [115].

Since the fossil record of lorisoids is deficient and only one appropriate calibration point is available for dating the lorisoid tree – the stem galagid *Saharagalago* dated between 36.9 and 42 Ma [39] – we also included four additional calibration points within primates: *Homo*/*Pan*, *Homo*/*Pongo*, divergence of crown catarrhines (split between Hominoidea and Cercopithecoidea), and *Theropithecus*/*Papio*. These four nodes are well supported by fossil evidence and have been commonly accepted as appropriate calibration points to date divergences within primates [75,76,124-128]. Calibration points were implemented as translated-lognormal distributions (*i.e.* lognormally distributed, with an offset roughly equal to the age of the fossil [129-132]). While minimum hard boundaries can be defined by the oldest known fossils bearing derived characters diagnostic of a clade [126,132-134], maximum bounds for a particular split are inherently unknowable based on fossil evidence (e.g. [132,135]). We therefore applied soft maximum bounds to account for uncertainty in the older limits. Details of the fossil evidence and parameters used to run dating analyses in BEAST are reported in Table 2.

**Table 2:** Evolutionary rate calibration constraints (in millions of years)

**Divergence**	**Offset**	**95% prior distribution**	**Mean**	**Fossil**		**Reference**	**Age**
1. *Homo/Pan*	5.0	10.0	2.5	*Ardipithecus*	[136]	5.2
				*Orrorin*	[137]	6.0
				*Sahelanthropus*	[138,139]	6.0-7.0
2. *Homo/Pongo*	12.5	18.0	2.75	*Sivapithecus*	[140]	≈12.5
3. Crown Catarrhini	21.0	33.9	6.43	*Morotopithecus*	[141]	>20.6
				*Victoriapithecus*	[142,143]	≈19.0
4. *Theropithecus/Papio*	3.5	6.5	1.5	*Theropithecus*	[144,145]	≈3.5
5. Crown Lorisoidea	36.9	47.0	5	*Saharagalago*	[39]	>36.9
				*Wadilemur*	[40]	≈35.0

Calibration points were treated as translated-lognormal distributions with hard offset and soft maximum bounds. Standard deviation was set at 0.5 for all nodes.

## Availability of supporting data

The data sets supporting the results of this article are available in the TreeBase repository, http://purl.org/phylo/treebase/phylows/study/TB2:S15281. A complete list of the new sequences generated for this study, including GenBank accession numbers is available in Additional File 5.

## Competing interests

The authors declare that they have no competing interests.

## Authors’ contributions

LP and JCM conceived the study and wrote the manuscript. LP and JCM obtained the samples. LP obtained and analyzed genetic data. LP and TRD obtained funding support for both field and lab components. All authors have read and approved the final manuscript.

## Supplementary Material

Additional file 1 **Support values for all the phylogenetic analyses conducted (RAxML, MrBayes, BEST, and BEAST) and date estimates with 95% highest probability densities (HPDs) for each node in the tree (Ga. = *Galago;* Gs. = *Galagoides*).** Click here for file

Additional file 2 Individual-locus Bayesian support for all the nodes strongly supported in the concatenated Bayesian analyses for the dataset 27LOCI (Table S2a) and 19LOCI (Table S2b). Click here for file

Additional file 3 **Phylogenetic trees inferred from coalescent-based species tree analyses performed using BEST v2.3 (a: 27LOCI and b: 19LOCI).** Numbers inside the white boxes indicate node numbers. Only posterior probabilities lower than 1.00 are reported in the figure. Click here for file

Additional file 4 **List of loci used in this study.** The table includes name of the loci, primer sequences, description based on the human genome, and reference for the primers. Click here for file

Additional file 5 List of the GenBank accession numbers for all the sequences included in the study. Click here for file

Additional file 6 List of genetic samples used in this study including specimen ID, source, and number of loci. Click here for file

Additional file 7 **List of the loci used in the coalescent-based species tree analyses for both datasets 27LOCI (Table S7a) and 19LOCI (Table S7b).** For each dataset we report name of the locus, length (bp), number and percentage of constant, variable and parsimony informative characters. Click here for file
