# Supplementary material for: A multilocus phylogeny reveals deep lineages within African galagids (Primates: Galagidae)
Source: BMC Evol Biol. 2014 Apr 2;14:72. doi: 10.1186/1471-2148-14-72 (PMC4021292; doi:10.1186/1471-2148-14-72)
Supplement: Additional file 1 — Support values for all the phylogenetic analyses conducted (RAxML, MrBayes, BEST, and BEAST) and date estimates with 95% highest probability densities (HPDs) for each node in the tree (Ga. = Galago; Gs. = Galagoides ). [file 1471-2148-14-72-S1.docx]

**Additional file 1 – Table S1**

Support values for all the phylogenetic analyses conducted (RAxML, MrBayes, BEST, and BEAST) and date estimates with 95% highest probability densities (HPDs) for each node in the tree (*Ga.=Galago*; *Gs.=Galagoides*).

|  | Split | RAxML (BP) | MrBayes (PP)* |  | BEST (PP)* | |  | BEAST | | | |
| --- | --- | --- | --- | --- | --- | --- | --- | --- | --- | --- | --- |
| Node |  |  |  |  | 27LOCI | 19LOCI |  | PP | Mean | *95% HPD* | |
|  |  |  |  |  |  |  |  |  |  | |  |
| 1 | Crown Lorisoidea | 100 | 1.00 |  | 1.00 | 1.00 |  | 1.00 | 41.25 | | 38.22-44.68 |
| 2 | Crown Lorisidae | 76 | --- |  | --- | --- |  | 0.86 | 39.92 | | 36.70-43.33 |
| * | Asian Lorisidae/Galagidae | --- | 0.70 |  | 0.73 | 0.65 |  | --- | --- | | --- |
| 3 | Crown Galagidae | 100 | 1.00 |  | 1.00 | 1.00 |  | 1.00 | 33.29 | | 29.96-36.82 |
| 4 | African Lorisidae | 100 | 1.00 |  | 1.00 | 1.00 |  | 1.00 | 22.59 | | 19.3-26.15 |
| 5 | Asian Lorisidae | 100 | 1.00 |  | 1.00 | 1.00 |  | 1.00 | 21.33 | | 18.44-24.36 |
| *6* | *Nycticebus* spp. | 100 | 1.00 |  | n/a | n/a |  | 1.00 | 6.78 | | 5.32-8.36 |
| *7* | *N. bengalensis-N. coucang* | 100 | 1.00 |  | n/a | n/a |  | 1.00 | 1.04 | | 0.53-1.63 |
| *8* | *Galagoides*-other galagids | 100 | 1.00 |  | 1.00 | 1.00 |  | 1.00 | 19.54 | | 17.29-21.87 |
| *9* | *Galagoides* spp. | 100 | 1.00 |  | 1.00 | n/a |  | 1.00 | 9.33 | | 7.67-11.09 |
| *10* | *Gs. thomasi/Gs. demidoff* #2 | 100 | 1.00 |  | n/a | n/a |  | 1.00 | 5.00 | | 3.66-6.44 |
| *11* | *Otolemur/Galago-‘Galagoides’* | 100 | 1.00 |  | 1.00 | 1.00 |  | 1.00 | 15.84 | | 13.93-17.85 |
| *12* | *Galago-‘Galagoides’* | 96 | 1.00 |  | 0.87 | 0.87 |  | 1.00 | 14.12 | | 12.34-16.09 |
| *13* | *Galago* spp. | 100 | 1.00 |  | 1.00 | 1.00 |  | 1.00 | 4.45 | | 3.51-5.52 |
| 14 | *Ga. moholi* #2- other *Galago* | 100 | 1.00 |  | 1.00 | 0.98 |  | 1.00 | 1.23 | | 0.77-1.75 |
| *15* | *Ga. moholi* #1 | 90 | 1.00 |  | n/a | n/a |  | 1.00 | 0.72 | | 0.42-1.03 |
| *16* | *Ga. moholi* #3/*Ga. senegalensis* | 76 | n/a |  | n/a | n/a |  | 0.42 | 0.61 | | 0.33-0.91 |
| *17* | *‘Gs.’ cocos/’Gs’. zanzibaricus* | 100 | 1.00 |  | 1.00 | 1.00 |  | 1.00 | 3.58 | | 2.57-4.63 |
| *18* | *Otolemur* spp. | 100 | 1.00 |  | 1.00 | n/a |  | 1.00 | 6.56 | | 5.06-8.09 |
| *19* | *O. g. garnettii/O. g. lasiotis* | 100 | 1.00 |  | n/a | n/a |  | 1.00 | 1.75 | | 1.11-2.46 |
|  |  |  |  |  |  |  |  |  |  | |  |

* The topology obtained from MrBayes and BEST analyses differ from the ML and BEAST analyses and support the paraphyletic status of Lorisidae, with Asian lorises sister group of galagids.
