# Supplementary material for: A multilocus phylogeny reveals deep lineages within African galagids (Primates: Galagidae)
Source: BMC Evol Biol. 2014 Apr 2;14:72. doi: 10.1186/1471-2148-14-72 (PMC4021292; doi:10.1186/1471-2148-14-72)
Supplement: Additional file 2 — Individual-locus Bayesian support for all the nodes strongly supported in the concatenated Bayesian analyses for the dataset 27LOCI (Table S2a) and 19LOCI (Table S2b). [file 1471-2148-14-72-S2.docx]

**Table S2a** Individual-locus Bayesian support for all the nodes supported in the BEST analyses using 27 loci

| Locus | Posterior probabilities or nodes strongly supported in Bayesian (MrBayes) concatenated analyses | | | | | | | | | | | | | | | | |
| --- | --- | --- | --- | --- | --- | --- | --- | --- | --- | --- | --- | --- | --- | --- | --- | --- | --- |
|  | 1 | 2A | 2B | 2C | 3 | 4 | 5 | 6 | 7 | 8 | 9A | 9B | 9C | 10 | 11 | 12 | 13 |
|  |  |  |  |  |  |  |  |  |  |  |  |  |  |  |  |  |  |
| ABCA1 | 1.00 | 0.54 | 0.00 | 0.00 | 1.00 | 0.93 | n/a | 1.00 | 1.00 | 1.00 | 0.00 | 0.00 | 0.28 | 1.00 | n/a | n/a | 1.00 |
| ADORA3 | 1.00 | 0.00 | 0.00 | 0.55 | n/a | 1.00 | 1.00 | 0.00 | 0.00 | 0.00 | 0.00 | 0.00 | 0.00 | 0.00 | 0.00 | 0.95 | 0.00 |
| AFF2 | 1.00 | 0.45 | 0.00 | 0.00 | 1.00 | 1.00 | 0.98 | 0.39 | 0.77 | 1.00 | 0.34 | 0.00 | 0.00 | 1.00 | 1.00 | 0.97 | 0.98 |
| APP | 1.00 | 0.00 | 0.00 | 0.35 | 0.00 | n/a | n/a | 0.00 | 0.00 | 0.00 | 0.00 | 0.00 | 0.00 | 0.00 | 0.16 | 0.00 | 1.00 |
| ATXN7 | 1.00 | 0.63 | 0.00 | 0.00 | 0.50 | 1.00 | n/a | 0.99 | 1.00 | 0.35 | 0.00 | 0.48 | 0.00 | 1.00 | 0.90 | 0.54 | 1.00 |
| AXIN1 | 1.00 | 0.00 | 0.00 | 0.74 | 0.67 | 1.00 | 1.00 | 0.99 | n/a | 0.77 | 0.78 | 0.00 | 0.00 | 0.91 | 1.00 | 0.00 | 0.96 |
| BCOR | 1.00 | 0.00 | 0.00 | 0.00 | 0.00 | 1.00 | 0.58 | 0.97 | n/a | 0.98 | 0.95 | 0.00 | 0.00 | 1.00 | 1.00 | 1.00 | 1.00 |
| CHRNA1 | 1.00 | 0.99 | 0.00 | 0.00 | 0.97 | 1.00 | 1.00 | 0.00 | 1.00 | 0.00 | 0.81 | 0.00 | 0.00 | 1.00 | 1.00 | 0.35 | 1.00 |
| DACH1 | 1.00 | 0.00 | 0.00 | 0.81 | 0.76 | 1.00 | 1.00 | 1.00 | 1.00 | 0.97 | 0.35 | 0.00 | 0.00 | n/a | 1.00 | 0.00 | 1.00 |
| DCTN2 | 1.00 | 0.00 | 0.00 | 0.00 | 0.00 | 0.99 | 0.98 | 1.00 | 1.00 | 1.00 | 0.00 | 0.00 | 0.34 | n/a | 0.99 | 0.00 | 1.00 |
| DENND5A | 1.00 | 0.00 | 0.00 | 0.82 | 0.83 | 1.00 | 1.00 | 1.00 | 1.00 | 0.68 | 0.98 | 0.00 | 0.00 | 1.00 | 1.00 | 0.00 | 1.00 |
| ERC2 | 1.00 | 0.00 | 1.00 | 0.00 | 1.00 | 1.00 | 1.00 | 1.00 | 1.00 | 0.99 | n/a | n/a | n/a | 1.00 | 1.00 | 0.94 | n/a |
| FAM123B | 1.00 | 0.00 | 0.00 | 0.00 | 0.00 | 1.00 | n/a | 0.79 | 0.82 | 0.79 | 0.79 | 0.00 | 0.00 | 0.33 | 0.80 | 0.79 | 1.00 |
| FBN1 | 1.00 | 0.81 | 0.00 | 0.00 | 1.00 | 0.99 | 1.00 | 0.99 | 1.00 | 0.20 | 0.00 | 0.20 | 0.00 | 0.94 | 1.00 | 0.00 | 1.00 |
| GHR | 1.00 | 0.00 | 0.00 | 0.87 | 1.00 | 1.00 | n/a | 1.00 | n/a | 0.61 | 0.00 | 0.00 | 1.00 | 1.00 | 0.98 | 1.00 | 1.00 |
| KCNMA1 | 1.00 | 0.00 | 0.00 | 0.37 | 0.76 | 0.94 | 1.00 | 0.00 | n/a | 0.85 | 0.00 | 0.00 | 0.44 | n/a | 1.00 | 1.00 | 1.00 |
| LRPPRC-171 | 1.00 | 0.00 | 0.00 | 0.68 | 0.87 | 1.00 | 1.00 | 1.00 | 1.00 | 1.00 | 0.00 | 0.79 | 0.00 | 0.97 | 1.00 | 0.85 | 1.00 |
| LUC7L | 1.00 | 0.00 | 0.00 | 0.53 | 1.00 | 1.00 | 1.00 | 1.00 | 0.96 | 0.00 | 0.00 | 0.00 | 0.20 | n/a | 1.00 | 0.99 | 1.00 |
| NPAS3.2 | 1.00 | 0.65 | 0.00 | 0.00 | 1.00 | 1.00 | 1.00 | 1.00 | 0.00 | 0.99 | 1.00 | 0.00 | 0.00 | 0.98 | 1.00 | 1.00 | 1.00 |
| PNOC | 1.00 | 0.00 | 0.00 | 0.00 | 0.00 | n/a | 0.97 | 0.00 | 0.30 | 0.48 | 0.00 | 0.00 | 0.00 | 0.00 | 0.75 | 0.00 | 0.99 |
| POLA1 | 1.00 | 0.71 | 0.00 | 0.00 | 0.73 | 0.75 | 1.00 | 0.82 | 0.99 | 0.78 | 0.00 | 0.00 | 0.49 | 1.00 | 0.82 | 0.89 | 0.88 |
| RAG2 | 1.00 | 0.00 | 0.00 | 0.34 | 0.68 | 0.99 | 1.00 | 0.90 | n/a | 0.42 | 0.00 | 0.00 | 0.17 | 0.21 | 0.39 | 0.00 | 0.86 |
| RPGRIP1 | 1.00 | 0.64 | 0.00 | 0.00 | 0.32 | 1.00 | 1.00 | 1.00 | n/a | 0.98 | 0.00 | 0.00 | 0.00 | 0.97 | 0.00 | 0.97 | 0.97 |
| SGMS1 | 1.00 | 0.00 | 0.00 | 0.37 | 0.74 | 0.93 | 1.00 | 0.94 | 0.66 | 0.00 | 0.00 | 0.00 | 0.00 | 0.00 | 1.00 | 0.34 | 0.00 |
| SIM1 | 1.00 | 0.00 | 0.00 | 0.00 | 0.00 | 1.00 | 1.00 | 0.96 | 0.00 | 0.00 | 0.00 | 0.00 | 0.16 | 0.98 | 1.00 | 1.00 | 0.98 |
| SMCX | 1.00 | 0.00 | 0.00 | 0.00 | 0.00 | 1.00 | 0.34 | 0.00 | 0.00 | 0.00 | 0.00 | 0.00 | 0.00 | 0.99 | 0.00 | 0.46 | 1.00 |
| ZIC3 | 1.00 | 0.00 | 0.26 | 0.00 | 0.57 | 0.58 | 1.00 | 1.00 | n/a | 0.00 | 0.00 | 0.00 | 0.00 | 0.00 | 0.00 | 0.00 | 0.94 |
| Mean | 1.00 | 0.20 | 0.05 | 0.24 | 0.59 | 0.96 | 0.95 | 0.73 | 0.68 | 0.55 | 0.23 | 0.06 | 0.12 | 0.71 | 0.76 | 0.54 | 0.91 |
| st. dev. | 0.00 | 0.33 | 0.20 | 0.32 | 0.40 | 0.10 | 0.16 | 0.42 | 0.43 | 0.42 | 0.38 | 0.18 | 0.23 | 0.43 | 0.39 | 0.44 | 0.27 |
|  |  |  |  |  |  |  |  |  |  |  |  |  |  |  |  |  |  |

Two independent runs of 40 million generations each were used for this analysis (10% of the trees were excluded). See numbered nodes from Additional file 3. Note: 1) Three possible topologies are reported for Node #2 (2A: Asian lorisids sister group of galagids; 2B: African lorisids sister group of galagids; 2C: lorisids form a monophyletic group); 2) Three possible topologies are reported for Node #9 (9A: *“Galagoides”* sister taxon of *Galago*; 9B: *Galago* sister taxon of *Otolemur*; and 9C: *“Galagoides”* sister taxon of *Otolemur*)

**Table S2b** Individual-locus Bayesian support for all the nodes supported in the BEST analyses using 19 loci.

| Locus | Posterior probabilities or nodes strongly supported in Bayesian (MrBayes) concatenated analyses | | | | | | | | | | | | | | | |
| --- | --- | --- | --- | --- | --- | --- | --- | --- | --- | --- | --- | --- | --- | --- | --- | --- |
|  | 1 | 2A | 2B | 2C | 3 | 4 | 5 | 6 | 7 | 8 | 9A | 9B | 9C | 10 | 11 | 12 |
|  |  |  |  |  |  |  |  |  |  |  |  |  |  |  |  |  |
| AFF2 | 1.00 | 0.46 | 0.00 | 0.00 | 1.00 | 1.00 | 0.99 | 0.95 | 0.99 | 1.00 | 0.00 | 0.00 | 0.38 | 1.00 | 0.98 | 0.99 |
| AXIN1 | 1.00 | 0.00 | 0.00 | 0.53 | 0.71 | 0.99 | 1.00 | 0.99 | 0.98 | 0.02 | 0.90 | 0.00 | 0.00 | 1.00 | 0.00 | 0.97 |
| BCOR | 1.00 | 0.00 | 0.00 | 0.00 | 0.00 | 1.00 | 0.00 | 0.97 | 0.77 | 0.67 | 0.97 | 0.00 | 0.00 | 1.00 | 1.00 | 1.00 |
| CHRNA1 | 1.00 | 0.93 | 0.00 | 0.00 | 0.89 | 1.00 | 1.00 | 0.87 | 0.97 | 0.78 | 0.00 | 0.73 | 0.00 | 1.00 | 0.34 | 1.00 |
| DACH1 | 1.00 | 0.00 | 0.00 | 0.84 | 0.82 | 1.00 | 1.00 | 1.00 | 1.00 | 0.97 | 0.34 | 0.00 | 0.00 | 1.00 | 0.34 | 1.00 |
| DCTN2 | 1.00 | 0.00 | 0.00 | 0.00 | 0.00 | 1.00 | 0.97 | 1.00 | 1.00 | 1.00 | 0.00 | 0.34 | 0.00 | 0.99 | 0.00 | 1.00 |
| DENND5A | 0.98 | 0.00 | 0.00 | 0.82 | 0.77 | 1.00 | 1.00 | 0.98 | 0.98 | 0.62 | 0.97 | 0.00 | 0.00 | 1.00 | 0.00 | 1.00 |
| FAM123B | 1.00 | 0.00 | 0.00 | 0.00 | 0.00 | 1.00 | 0.00 | 1.00 | 1.00 | 1.00 | 1.00 | 0.00 | 0.00 | 1.00 | 0.94 | 1.00 |
| FBN1 | 1.00 | 0.88 | 0.00 | 0.00 | 1.00 | 1.00 | 1.00 | 1.00 | 1.00 | 0.00 | 0.00 | 0.00 | 0.00 | 1.00 | 0.34 | 1.00 |
| KCNMA1 | 1.00 | 0.00 | 0.70 | 0.00 | 1.00 | 0.98 | 1.00 | 0.00 | 1.00 | 0.92 | 0.37 | 0.00 | 0.00 | 1.00 | 1.00 | 1.00 |
| LRPPRC-171 | 1.00 | 0.00 | 0.00 | 0.70 | 0.88 | 1.00 | 1.00 | 1.00 | 1.00 | 1.00 | 0.00 | 0.81 | 0.00 | 1.00 | 0.38 | 1.00 |
| LUC7L | 1.00 | 0.00 | 0.00 | 0.54 | 1.00 | 1.00 | 1.00 | 1.00 | 0.93 | 0.00 | 0.00 | 0.00 | 0.20 | 1.00 | 0.99 | 1.00 |
| NPAS3.2 | 1.00 | 0.66 | 0.00 | 0.00 | 1.00 | 1.00 | 1.00 | 1.00 | 0.00 | 0.99 | 1.00 | 0.00 | 0.00 | 0.98 | 1.00 | 1.00 |
| POLA1 | 1.00 | 0.90 | 0.00 | 0.00 | 0.91 | 0.91 | 1.00 | 1.00 | 1.00 | 0.94 | 0.00 | 0.00 | 0.63 | 1.00 | 1.00 | 1.00 |
| RAG2 | 1.00 | 0.36 | 0.00 | 0.00 | 0.98 | 1.00 | 1.00 | 1.00 | 1.00 | 0.92 | 0.00 | 0.00 | 0.49 | 0.91 | 0.84 | 0.97 |
| RPGRIP1 | 1.00 | 0.73 | 0.00 | 0.00 | 0.30 | 1.00 | 1.00 | 1.00 | 1.00 | 1.00 | 0.00 | 0.00 | 0.00 | 0.00 | 0.98 | 0.98 |
| SGMS1 | 1.00 | 0.00 | 0.00 | 0.34 | 0.93 | 0.99 | 1.00 | 1.00 | 0.72 | 0.00 | 0.00 | 0.15 | 0.00 | 1.00 | 0.00 | 0.00 |
| SIM1 | 1.00 | 0.00 | 0.22 | 0.00 | 0.00 | 1.00 | 1.00 | 0.97 | 0.00 | 0.00 | 0.00 | 0.00 | 0.00 | 1.00 | 1.00 | 0.99 |
| ZIC3 | 1.00 | 0.00 | 0.28 | 0.00 | 0.64 | 0.66 | 1.00 | 1.00 | 0.96 | 0.00 | 0.00 | 0.00 | 0.00 | 0.00 | 0.00 | 0.97 |
|  |  |  |  |  |  |  |  |  |  |  |  |  |  |  |  |  |
| Mean | 1.00 | 0.26 | 0.06 | 0.20 | 0.68 | 0.98 | 0.89 | 0.93 | 0.86 | 0.62 | 0.29 | 0.11 | 0.09 | 0.89 | 0.59 | 0.94 |
| st. dev. | 0.00 | 0.37 | 0.17 | 0.32 | 0.40 | 0.08 | 0.31 | 0.23 | 0.31 | 0.45 | 0.43 | 0.25 | 0.19 | 0.31 | 0.44 | 0.23 |
|  |  |  |  |  |  |  |  |  |  |  |  |  |  |  |  |  |

Two independent runs of 40 million generations each were used for this analysis (10% of the trees were excluded). See numbered nodes from Additional file 3.

Note: 1) Three possible topologies are reported for Node #2 (2A: Asian lorisids sister group of galagids; 2B: African lorisids sister group of galagids; 2C: lorisids form a monophyletic group); 2) Three possible topologies are reported for Node #9 (9A: *“Galagoides”* sister taxon of *Galago*; 9B: *Galago* sister taxon of *Otolemur*; and 9C: *“Galagoides”* sister taxon of *Otolemur*)
