# Supplementary material for: A multilocus phylogeny reveals deep lineages within African galagids (Primates: Galagidae)
Source: BMC Evol Biol. 2014 Apr 2;14:72. doi: 10.1186/1471-2148-14-72 (PMC4021292; doi:10.1186/1471-2148-14-72)
Supplement: Additional file 3 — Phylogenetic trees inferred from coalescent-based species tree analyses performed using BEST v2.3 (a: 27LOCI and b: 19LOCI). Numbers inside the white boxes indicate node numbers. Only posterior probabilities lower than 1.00 are reported in the figure. [file 1471-2148-14-72-S3.docx]

**Additional file 3. Phylogenetic trees inferred from coalescent-based species tree analyses performed using BEST v2.3 (a: 27LOCI and b: 19LOCI).** Numbers inside the white boxes indicate node numbers. Only posterior probabilities lower than 1.00 are reported in the figure.
