# Supplementary material for: A multilocus phylogeny reveals deep lineages within African galagids (Primates: Galagidae)
Source: BMC Evol Biol. 2014 Apr 2;14:72. doi: 10.1186/1471-2148-14-72 (PMC4021292; doi:10.1186/1471-2148-14-72)
Supplement: Additional file 4 — List of loci used in this study. The table includes name of the loci, primer sequences, description based on the human genome, and reference for the primers. [file 1471-2148-14-72-S4.docx]

| **Locus name** | **Full name** | **Forward primer sequence** | **Reverse primer sequence** | **Description Based on Human Genome** | **Reference** |
| --- | --- | --- | --- | --- | --- |
| ABCA1 | ATP-binding cassette, sub-family A (ABC1), member 1 | CCTCCATCTTTTCAGCTCTACCTAC | ACAAGAGCCTGGAGATTGGATAAC | Intronic | Horvath et al., 2008 |
| ADORA3 | adenosine A3 receptor | ACCCCCATGTTTGGCTGGAA | GATAGGGTTCATCATGGAGTT | Exonic | Murphy et al., 2001 |
| AFF2 | AF4/FMR2 family, member 2 | GCAGATTTGACATGATTGTGC | CAGTCAGGGAGGCCTTAAAA | Intronic | Perelman et al., 2011 |
| APP | amyloid beta (A4) precursor protein | TCCAAGATGCAGCAGAACG | CTAATGTGTGCACATAAAACAGG | Non coding region, one primer anchored in exon | Perelman et al., 2011 |
| ATXN7 | ataxin 7 | TGATCTGCCAGGACCTTTGT | TTGGCCAGA TCACTCAAA TG | Intronic | Perelman et al., 2011 |
| AXIN1 | axin 1 | CTCTGCCTTCGCTGTACCGTCTAC | GACCCACCTTTCCTAATCCTTGTC | Mostly Exonic | Horvath et al., 2008 |
| BCOR | BCL6 corepressor | AATCTCACTTCCGGAGAGCA | CCTTGCTACCTCAGCAGTCC | Exonic | Perelman et al., 2011 |
| CHRNA1 | cholinergic receptor, nicotinic, alpha 1 (muscle) | GACCATGAAGTCAGACCAGGAG | GGAGTATGTGGTCCATCACCAT | Mostly intronic, one primer anchored in exon | Lyons et al., 1997 |
| DACH1 | dachshund homolog 1 (Drosophila) | TAGCCAGCCACTGTGAGAGA | TGACTGATTACTGTCCTCGGG | Intronic | Perelman et al., 2011 |
| DCTN2 | dynactin 2 (p50) | TGGCTCTGGCTCTGTACTCA | GAAAACCTGGCCACAGTTGA | Mostly intronic, one primer anchored in exon | Perelman et al., 2011 |
| DENND5A | DENN/MADD domain containing 5A | CCAGAGTTATCATGGCCAATC | GTACCAAGCAAGAAGCTGGG | Mostly intronic, primers anchored in exons | Perelman et al., 2011 |
| ERC2 | ELKS/RAB6-interacting/CAST family member 2 | AGCTCATCCTCCTCCTGGTTTAG | CTCCTTGAGGATCTCCAGCAAC | Intronic | Horvath et al., 2008 |
| FAM123B | family with sequence similarity 123B | CATCACTCTGGAAGAGCTGC | TGGATTTGAGGATGATTCAGG | Exonic | Perelman et al., 2011 |
| FBN1 | fibrillin 1 | AGACTACCTCAGTGGTGAACTGG | AAGCACCATTACAAACCCTCA | Non coding region, one primer anchored in exon | Janecka et al., 2007 |
| GHR | growth hormone receptor | CCAGTTCCAGTTCCAAAGAT | TGATTCTTCTGGTCAAGGCA | Mostly intronic, one primer anchored in exon | Venta et al., 1996 |
| KCNMA1 | potassium large conductance calcium- activated channel, subfamily M, alpha member 1 | AAGTGGGAAGGCTCTCTCAA | CTCTGCCTAATGCCCAGAA | Intronic | Perelman et al., 2011 |
| LRPPRC-171 | leucine-rich PPR-motif containing | GACACAGGGAGTCTACACAAACAC | GATGCCAGAGCTCTCCTACAG | Mostly intronic, one primer anchored in exon | Horvath et al., 2008 |
| LUC7L | LUC7-like (S. cerevisiae) | CAGCAGTCCAGAAGGTGACTCTTG | GCAGATGGGTACAGTATGTGTTGG | Mostly intronic, one primer anchored in exon | Horvath et al., 2008 |
| NPAS3.2 | neuronal PAS domain protein 3 | TCAGCATTGTTGATCTGCTTTT | TGGAATATCTAACCATCTCTGAACA | Intronic | Perelman et al., 2011 |
| PNOC | prepronociceptin | GCATCCTTGAGTGTGAAGAGAA | TGCCTCATAAACTCACTGAACC | Exonic | Murphy et al., 2001 |
| POLA1 | polymerase (DNA directed), alpha 1, catalytic subunit | TGACAGTTGACAGACAAAGCAA | TGCCATCAGTTTGCTGAAAG | Intronic | Perelman et al., 2011 |
| RAG2 | recombination activating gene 2 | GATTCCTGCTAYCTYCCTCCTCT | CCCATGTTGCTTCCAAACCATA | Exonic | Teeling et al., 2000 |
| RPGRIP1 | retinitis pigmentosa GTPase regulator interacting protein 1 | AGATGTTGCTTATGGCACCC | ACCTGGGCTTTCTTTCGTTT | Exonic | Perelman et al., 2011 |
| SGMS1 | sphingomyelin synthase 1 | TCAGAATCAAACCCCATTCAG | GTGGTGGTACAGGCCATTTC | Mostly non coding region, one primer anchored in exon | Perelman et al., 2011 |
| SIM1 | single-minded homolog 1 (Drosophila) | GACCTACCGCAGAAAATTCG | CTGGGGCTCATCATTCA TTC | Intronic | Perelman et al., 2011 |
| SMCX | lysine (K)-speciVic demethylase 5C | GGAGGAGCTGAGACAGCTAGA | TGTACAACCCCAGCTCCTTC | Intronic, primers anchored in exons | Perelman et al., 2011 |
| ZIC3 | Zic family member 3 (odd-paired homolog, Drosophila) | ACCCGCTATAGCTTCTGCAA | ATAACAGGACTTTGGCACGG | Mostly non coding region, one primer anchored in exon | Perelman et al., 2011 |
