# Supplementary material for: A multilocus phylogeny reveals deep lineages within African galagids (Primates: Galagidae)
Source: BMC Evol Biol. 2014 Apr 2;14:72. doi: 10.1186/1471-2148-14-72 (PMC4021292; doi:10.1186/1471-2148-14-72)
Supplement: Additional file 5 — List of the GenBank accession numbers for all the sequences included in the study. [file 1471-2148-14-72-S5.docx]

**Table S5** List of the GenBank accession numbers for all the sequences used in this study. In bold are the sequences newly generated for this study.

|  | *LOCUS* | | | | | | | | | | | | | |
| --- | --- | --- | --- | --- | --- | --- | --- | --- | --- | --- | --- | --- | --- | --- |
| *SPECIMEN* | *ABCA1* | *ADORA3* | *AFF2* | *APP* | *ATXN7* | *AXIN1* | *BCOR* | *CHRNA1* | *DACH1* | *DCTN2* | *DENND5A* | *ERC2* | *FAM123B* | *FBN1* |
| ***Galagoides_demidoff*_3048f** | **KJ419042** | **KJ419047** | **KJ419058** | **---** | **KJ419075** | **KJ419086** | **KJ419095** | **KJ419100** | **KJ419110** | **KJ419117** | **KJ419127** | **KJ419135** | **KJ419143** | **KJ419158** |
| ***Galago_moholi*_ABSHER009f** | **KJ419046** | **KJ419051** | **KJ419055** | **KJ419069** | **KJ419078** | **KJ419083** | **KJ419094** | **KJ419103** | **KJ419113** | **KJ419123** | **KJ419132** | **KJ419138** | **KJ419147** | **KJ419154** |
| ***Galagoides_demidoff*_AMNH_269853** | **KJ419045** | **KJ419048** | **KJ419059** | **KJ419070** | **KJ419079** | **KJ419080** | **KJ419089** | **KJ419104** | **KJ419109** | **KJ419122** | **KJ419129** | **KJ419140** | **KJ419144** | **KJ419151** |
| ***Euoticus_elegantulus*_AMNH_269911** | **KJ419044** | **---** | **KJ419056** | **KJ419068** | **KJ419072** | **KJ419084** | **KJ419097** | **KJ419098** | **KJ419111** | **KJ419118** | **KJ419128** | **KJ419136** | **KJ419148** | **KJ419156** |
| ***Galago_matschiei*_FMNH_148985** | **---** | **KJ419049** | **KJ419060** | **KJ419064** | **KJ419073** | **KJ419085** | **KJ419091** | **KJ419102** | **KJ419115** | **KJ419121** | **KJ419133** | **KJ419139** | **KJ419149** | **KJ419152** |
| ***Galagoides_cocos*_GC-DN-006** | **KJ419040** | **KJ419052** | **KJ419061** | **KJ419065** | **KJ419077** | **KJ419081** | **KJ419092** | **KJ419101** | **KJ419114** | **KJ419119** | **KJ419131** | **---** | **KJ419141** | **KJ419153** |
| **G*alagoides_zanzibaricus*_GZ-UD-002** | **KJ419043** | **KJ419054** | **KJ419062** | **KJ419067** | **KJ419074** | **KJ419088** | **KJ419096** | **KJ419105** | **KJ419112** | **KJ419124** | **KJ419130** | **---** | **KJ419145** | **KJ419150** |
| ***Galago_moholi*_JCM001** | **---** | **KJ419050** | **---** | **KJ419063** | **KJ419071** | **KJ419087** | **KJ419090** | **KJ419099** | **KJ419108** | **KJ419120** | **KJ419126** | **KJ419134** | **KJ419146** | **KJ419155** |
| ***Otolemur_garnettii*_OG-DN-006** | **KJ419041** | **KJ419053** | **KJ419057** | **KJ419066** | **KJ419076** | **KJ419082** | **KJ419093** | **KJ419106** | **KJ419107** | **KJ419116** | **KJ419125** | **KJ419137** | **KJ419142** | **KJ419157** |
| *Arctocebus_calabarensis* ACL-1 | HM765259 | HM765244 | HM764896 | --- | HM764389 | HM764377 | HM764057 | HM763489 | HM762851 | HM762838 | HM759351 | HM762331 | HM762164 | HM761987 |
| *Galago_moholi*_GB GMO-4 | --- | --- | HM764969 | HM764708 | HM764449 | HM764379 | HM764059 | --- | HM762911 | HM762840 | HM759353 | --- | HM762166 | HM761989 |
| *Galago_senegalensis* GSE-1 | --- | HM765246 | HM764970 | HM764709 | HM764450 | HM764380 | HM764060 | HM763491 | HM762912 | HM762841 | HM759354 | HM762255 | HM762167 | HM761990 |
| *Galagoides_thomasi* GTH-1 | HM765320 | HM765247 | HM764971 | HM764710 | HM764451 | --- | --- | HM763492 | HM762913 | HM762842 | HM759355 | HM762298 | HM762168 | HM761991 |
| *Loris_tardigradus* LTA-2 | --- | HM765248 | HM764987 | HM764711 | --- | HM764381 | HM764061 | HM763493 | HM762929 | HM762843 | HM759356 | HM762194 | HM762169 | HM761992 |
| *Nycticebus_bengalensis* NBE-1 | HM765358 | HM765249 | HM765008 | HM764712 | HM764486 | HM764382 | HM764062 | HM763494 | HM762948 | HM762844 | HM759357 | HM762300 | HM762170 | HM761993 |
| *Nycticebus_coucang* NCO-2 | HM765360 | HM765250 | HM765010 | --- | HM764488 | HM764383 | HM764063 | HM763495 | HM762950 | HM762845 | HM759358 | HM762299 | --- | HM761994 |
| *Nycticebus_pygmaeus* NPY-1 | HM765363 | HM765251 | HM765013 | HM764713 | HM764491 | HM764384 | HM764064 | HM763496 | HM762953 | HM762846 | HM759359 | HM762195 | HM762171 | HM761995 |
| *Otolemur_crassicaudatus* OCR-1 | HM765365 | HM765243 | HM765015 | HM764706 | HM764493 | HM764376 | HM764056 | HM763488 | --- | --- | HM759350 | HM762301 | HM762163 | HM761986 |
| *Otolemur_garnetti* GGR-2 | --- | HM765245 | HM764968 | HM764707 | HM764448 | HM764378 | HM764058 | HM763490 | HM762910 | HM762839 | HM759352 | HM762244 | HM762165 | HM761988 |
| *Perodicticus_potto* PEP-2 | HM765372 | HM765252 | HM765021 | HM764714 | HM764500 | HM764385 | HM764065 | HM763497 | HM762961 | HM762847 | HM759360 | HM762223 | HM762172 | HM761996 |
| *Lemur_catta* LCT-10 | HM765333 | HM765237 | HM764981 | HM764700 | HM764464 | HM764371 | HM764050 | HM763484 | HM762925 | HM762832 | HM759344 | HM762277 | HM762157 | HM761981 |
| *Propithecus_verreauxi* PVE-1 | HM765386 | HM765225 | HM765036 | HM764689 | HM764514 | HM764366 | HM764044 | HM763478 | HM762975 | HM762821 | HM759333 | HM762270 | HM762146 | HM761971 |
| *Daubentonia_madagascariensis* DMD-5 | --- | --- | HM764960 | HM764680 | HM764440 | --- | --- | --- | HM762902 | HM762812 | HM759326 | --- | --- | HM761963 |
| *Homo_sapiens* HAS-34 | HM765327 | HM765141 | HM764976 | HM764615 | HM764458 | HM764284 | HM763963 | HM763397 | HM762920 | HM762733 | HM759236 | HM762263 | HM762062 | HM761896 |
| *Macaca_mulatta* MMA-14 | HM765347 | HM765108 | HM764996 | HM764581 | HM764474 | HM764251 | HM763929 | HM763365 | HM762937 | HM762716 | HM759202 | HM762276 | HM762029 | HM761863 |
| *Chlorocebus_aethiops* CAE-4 | HM765274 | HM765095 | HM764915 | HM764569 | HM764405 | HM764238 | HM763915 | HM763352 | HM762866 | HM762703 | HM759189 | HM762226 | HM762015 | HM761849 |
| *Pan_troglodytes* PTR-104 | HM765384 | HM765153 | HM765033 | HM764627 | HM764511 | HM764294 | HM763974 | HM763408 | HM762972 | HM762745 | HM759248 | HM762264 | HM762073 | HM761907 |
| *Papio_hamadryas* PHM-1 | HM765373 | HM765122 | HM765022 | HM764595 | HM764501 | HM764265 | HM763943 | HM763378 | HM762962 | HM762729 | HM759216 | HM762222 | HM762042 | HM761877 |
| *Pongo_pygmaeus* PPY-155 | HM765381 | HM765155 | HM765030 | HM764629 | HM764508 | HM764296 | HM763975 | HM763410 | HM762969 | HM762747 | HM759250 | HM762206 | HM762076 | HM761909 |
| *Theropithecus_gelada* TGE-2 | HM765407 | HM765133 | HM765060 | HM764607 | --- | HM764276 | HM763955 | HM763389 | HM762997 | HM762732 | HM759228 | HM762318 | HM762054 | --- |

**Table S5 (continued)**

|  | *LOCUS* | | | | | | | | | | | | |
| --- | --- | --- | --- | --- | --- | --- | --- | --- | --- | --- | --- | --- | --- |
| *SPECIMEN* | GHR | KCNMA1 | SMCX | LRPPRC-171 | LUC7L | NPAS3.2 | PNOC | POLA1 | RAG2 | RPGRIP1 | SGMS1 | SIM1 | ZIC3 |
| ***Galagoides_demidoff*_3048f** | **---** | **KJ419173** | **KJ419180** | **KJ419190** | **KJ419197** | **KJ419207** | **KJ419216** | **KJ419219** | **KJ419228** | **KJ419238** | **KJ419250** | **KJ419256** | **KJ419269** |
| ***Galago_moholi*_ABSHER009f** | **KJ419161** | **KJ419171** | **KJ419177** | **KJ419192** | **KJ419200** | **KJ419204** | **KJ419214** | **KJ419220** | **KJ419232** | **KJ419243** | **KJ419248** | **KJ419259** | **KJ419268** |
| ***Galagoides_demidoff*_AMNH_269853** | **KJ419163** | **KJ419167** | **KJ419182** | **KJ419186** | **KJ419196** | **KJ419205** | **KJ419211** | **KJ419224** | **KJ419229** | **KJ419242** | **KJ419251** | **KJ419257** | **KJ419265** |
| ***Euoticus_elegantulus*_AMNH_269911** | **KJ419160** | **KJ419174** | **KJ419176** | **KJ419185** | **KJ419195** | **KJ419208** | **KJ419212** | **KJ419221** | **KJ419230** | **KJ419241** | **KJ419253** | **KJ419260** | **KJ419270** |
| ***Galago_matschiei*_FMNH_148985** | **KJ419165** | **KJ419172** | **KJ419179** | **KJ419189** | **KJ419201** | **KJ419202** | **KJ419218** | **KJ419225** | **KJ419236** | **KJ419244** | **KJ419246** | **KJ419261** | **KJ419266** |
| ***Galagoides_cocos*_GC-DN-006** | **KJ419166** | **KJ419169** | **KJ419183** | **KJ419191** | **KJ419194** | **KJ419203** | **KJ419217** | **KJ419223** | **KJ419235** | **KJ419240** | **KJ419254** | **KJ419263** | **KJ419267** |
| **G*alagoides_zanzibaricus*_GZ-UD-002** | **KJ419159** | **KJ419168** | **KJ419178** | **KJ419184** | **KJ419199** | **KJ419206** | **KJ419215** | **KJ419227** | **KJ419234** | **KJ419239** | **KJ419249** | **KJ419262** | **KJ419264** |
| ***Galago_moholi*_JCM001** | **KJ419164** | **---** | **KJ419175** | **KJ419187** | **KJ419193** | **KJ419210** | **---** | **KJ419222** | **KJ419233** | **KJ419237** | **KJ419252** | **KJ419258** | **KJ419271** |
| ***Otolemur_garnettii*_OG-DN-006** | **KJ419162** | **KJ419170** | **KJ419181** | **KJ419188** | **KJ419198** | **KJ419209** | **KJ419213** | **KJ419226** | **KJ419231** | **KJ419245** | **KJ419247** | **KJ419255** | **KJ419272** |
| *Arctocebus_calabarensis* ACL-1 | HM761376 | HM761198 | HM758278 | HM761061 | HM760777 | HM759889 | HM759706 | HM759365 | HM759000 | HM758643 | HM758467 | HM758291 | HM756902 |
| *Galago_moholi*_GB GMO-4 | --- | --- | HM758280 | --- | HM760828 | HM759959 | HM759708 | HM759435 | HM759002 | --- | HM758528 | HM758354 | HM756904 |
| *Galago_senegalensis* GSE-1 | HM761378 | HM761253 | HM758281 | HM761063 | HM760829 | HM759960 | HM759709 | HM759436 | HM759003 | HM758729 | HM758529 | HM758355 | HM756905 |
| *Galagoides_thomasi* GTH-1 | --- | --- | HM758282 | HM761064 | HM760830 | HM759961 | HM759710 | HM759437 | --- | --- | HM758530 | HM758356 | --- |
| *Loris_tardigradus* LTA-2 | --- | HM761276 | HM758283 | HM761065 | HM760848 | HM759980 | HM759711 | HM759456 | HM759004 | HM758748 | HM758552 | HM758373 | HM756906 |
| *Nycticebus_bengalensis* NBE-1 | HM761379 | HM761302 | --- | --- | HM760871 | HM760001 | HM759712 | HM759477 | HM759005 | HM758769 | HM758580 | HM758393 | HM756907 |
| *Nycticebus_coucang* NCO-2 | HM761380 | HM761303 | HM758284 | HM761066 | HM760872 | HM760003 | HM759713 | HM759478 | HM759006 | HM758771 | HM758581 | HM758395 | HM756908 |
| *Nycticebus_pygmaeus* NPY-1 | --- | HM761304 | --- | HM761067 | HM760873 | HM760006 | HM759714 | HM759481 | HM759007 | HM758774 | HM758582 | HM758398 | HM756909 |
| *Otolemur_crassicaudatus* OCR-1 | HM761375 | --- | HM758277 | HM761060 | --- | HM760008 | HM759705 | HM759483 | HM758999 | HM758776 | HM758583 | HM758400 | HM756901 |
| *Otolemur_garnetti* GGR-2 | --- | HM761252 | HM758279 | HM761062 | HM760827 | HM759958 | HM759707 | HM759434 | HM759001 | HM758728 | HM758527 | HM758353 | HM756903 |
| *Perodicticus_potto* PEP-2 | HM761381 | HM761311 | HM758285 | HM761068 | HM760880 | HM760015 | --- | HM759490 | HM759008 | HM758783 | HM758590 | HM758406 | HM756910 |
| *Lemur_catta* LCT-10 | HM761370 | HM761267 | HM758271 | HM761055 | HM760840 | HM759974 | HM759699 | HM759450 | HM758993 | HM758742 | HM758543 | HM758368 | HM756896 |
| *Propithecus_verreauxi* PVE-1 | HM761361 | HM761320 | HM758261 | HM761048 | HM760890 | HM760029 | HM759688 | HM759504 | HM758983 | HM758799 | HM758602 | HM758421 | HM756887 |
| *Daubentonia_madagascariensis* DMD-5 | --- | --- | HM758252 | --- | --- | HM759951 | HM759681 | HM759427 | HM758974 | HM758720 | HM758521 | --- | HM756878 |
| *Homo_sapiens* HAS-34 | HM761441 | HM761257 | HM758189 | HM760985 | HM760834 | HM759968 | HM759608 | HM759444 | HM758896 | HM758736 | HM758534 | HM758362 | HM756808 |
| *Macaca_mulatta* MMA-14 | HM761424 | HM761282 | HM758156 | HM760951 | HM760854 | HM759989 | HM759577 | HM759465 | HM758862 | HM758758 | HM758558 | HM758381 | HM756774 |
| *Chlorocebus_aethiops* CAE-4 | HM761421 | HM761239 | HM758143 | HM760937 | HM760816 | HM759908 | HM759563 | HM759384 | HM758848 | HM758667 | HM758513 | HM758308 | HM756760 |
| *Pan_troglodytes* PTR-104 | HM761452 | HM761306 | HM758193 | HM760995 | HM760875 | HM760027 | HM759619 | HM759501 | HM758908 | HM758796 | HM758586 | HM758418 | HM756819 |
| *Papio_hamadryas* PHM-1 | HM761401 | HM761309 | HM758170 | HM760965 | HM760878 | HM760016 | HM759591 | HM759491 | HM758876 | HM758784 | HM758588 | HM758407 | HM756788 |
| *Pongo_pygmaeus* PPY-155 | HM761453 | HM761314 | HM758195 | HM760996 | HM760884 | HM760024 | HM759622 | HM759498 | HM758909 | HM758793 | HM758596 | HM758415 | HM756821 |
| *Theropithecus_gelada* TGE-2 | HM761403 | HM761343 | HM758182 | HM760977 | --- | HM760051 | --- | HM759527 | HM758888 | HM758824 | HM758628 | --- | HM756800 |
