# Supplementary material for: A multilocus phylogeny reveals deep lineages within African galagids (Primates: Galagidae)
Source: BMC Evol Biol. 2014 Apr 2;14:72. doi: 10.1186/1471-2148-14-72 (PMC4021292; doi:10.1186/1471-2148-14-72)
Supplement: Additional file 6 — List of genetic samples used in this study including specimen ID, source, and number of loci. [file 1471-2148-14-72-S6.docx]

**Table S5.** List of the specimens and number of loci used in this study

| Species | | Specimen ID | Specimen source | Loci | | | | | | | | | | | | | | | | | | | | | | | | | | | |
| --- | --- | --- | --- | --- | --- | --- | --- | --- | --- | --- | --- | --- | --- | --- | --- | --- | --- | --- | --- | --- | --- | --- | --- | --- | --- | --- | --- | --- | --- | --- | --- |
|  |  |  |  | TOTAL NUMBER | ABCA1 | ADORA3 | AFF2 | APP | ATXN7 | AXIN1 | BCOR | CHRNA1 | DACH1 | DCTN2 | DENND5A | ERC2 | FAM123B | FBN1 | GHR | KCNMA1 | LRPPRC-171 | LUC7L | NPAS3.2 | PNOC | POLA1 | RAG2 | RPGRIP1 | SGMS1 | SIM1 | SMCX | ZIC3 |
| *GALAGIDAE* | *Euoticus elegantulus* | AMNH_269911 | Dzanga Sangha FR, Central African Republic | 26 |  |  |  |  |  |  |  |  |  |  |  |  |  |  |  |  |  |  |  |  |  |  |  |  |  |  |  |
|  | *Galagoides thomasi* | GTH-1 | GenBank (Perelman et al., 2011) | 20 |  |  |  |  |  |  |  |  |  |  |  |  |  |  |  |  |  |  |  |  |  |  |  |  |  |  |  |
|  | *Galagoides demidoff* (#1) | AMNH_269853 | Dzanga Sangha FR, Central African Republic | 27 |  |  |  |  |  |  |  |  |  |  |  |  |  |  |  |  |  |  |  |  |  |  |  |  |  |  |  |
|  | *Galagoides demidoff* (#2) | 3048f | Cameroon | 25 |  |  |  |  |  |  |  |  |  |  |  |  |  |  |  |  |  |  |  |  |  |  |  |  |  |  |  |
|  | *Galagoides cocos* | GC-DN-006 | Diani Forest, Kenya | 26 |  |  |  |  |  |  |  |  |  |  |  |  |  |  |  |  |  |  |  |  |  |  |  |  |  |  |  |
|  | *Galagoides zanzibaricus* | GZ-UD-002 | Udzungwa NP, Tanzania | 26 |  |  |  |  |  |  |  |  |  |  |  |  |  |  |  |  |  |  |  |  |  |  |  |  |  |  |  |
|  | *Galago matschiei* | FMNH_148985 | Kibara National Park, Burundi | 26 |  |  |  |  |  |  |  |  |  |  |  |  |  |  |  |  |  |  |  |  |  |  |  |  |  |  |  |
|  | *Galago moholi* (#1) | JCM001 | Gauteng, South Africa | 23 |  |  |  |  |  |  |  |  |  |  |  |  |  |  |  |  |  |  |  |  |  |  |  |  |  |  |  |
|  | *Galago moholi* (#2) | GMO-4 | GenBank (Perelman et al., 2011) | 19 |  |  |  |  |  |  |  |  |  |  |  |  |  |  |  |  |  |  |  |  |  |  |  |  |  |  |  |
|  | *Galago moholi* (#3) | ABSHER009f | Duke University Lemur Center | 27 |  |  |  |  |  |  |  |  |  |  |  |  |  |  |  |  |  |  |  |  |  |  |  |  |  |  |  |
|  | *Galago senegalensis* | GSE-1 | GenBank (Perelman et al., 2011) | 26 |  |  |  |  |  |  |  |  |  |  |  |  |  |  |  |  |  |  |  |  |  |  |  |  |  |  |  |
|  | *Otolemur garnettii* | GGR-2 | GenBank (Perelman et al., 2011) | 25 |  |  |  |  |  |  |  |  |  |  |  |  |  |  |  |  |  |  |  |  |  |  |  |  |  |  |  |
|  | *Otolemur garnettii* | OG-DN-006 | Diani Forest, Kenya | 27 |  |  |  |  |  |  |  |  |  |  |  |  |  |  |  |  |  |  |  |  |  |  |  |  |  |  |  |
|  | *Otolemur crassicaudatus* | OCR-1 | GenBank (Perelman et al., 2011) | 23 |  |  |  |  |  |  |  |  |  |  |  |  |  |  |  |  |  |  |  |  |  |  |  |  |  |  |  |
| *LORISIDAE* | *Perodicticus potto* | PEP-2 | GenBank (Perelman et al., 2011) | 26 |  |  |  |  |  |  |  |  |  |  |  |  |  |  |  |  |  |  |  |  |  |  |  |  |  |  |  |
|  | *Arctocebus calabarensis* | ACL-1 | GenBank (Perelman et al., 2011) | 26 |  |  |  |  |  |  |  |  |  |  |  |  |  |  |  |  |  |  |  |  |  |  |  |  |  |  |  |
|  | *Nycticebus pygmaeus* | NPY-1 | GenBank (Perelman et al., 2011) | 25 |  |  |  |  |  |  |  |  |  |  |  |  |  |  |  |  |  |  |  |  |  |  |  |  |  |  |  |
|  | *Nycticebus bengalensis* | NBE-1 | GenBank (Perelman et al., 2011) | 25 |  |  |  |  |  |  |  |  |  |  |  |  |  |  |  |  |  |  |  |  |  |  |  |  |  |  |  |
|  | *Nycticebus coucang* | NCO-2 | GenBank (Perelman et al., 2011) | 25 |  |  |  |  |  |  |  |  |  |  |  |  |  |  |  |  |  |  |  |  |  |  |  |  |  |  |  |
|  | *Loris tardigradus* | LTA-2 | GenBank (Perelman et al., 2011) | 24 |  |  |  |  |  |  |  |  |  |  |  |  |  |  |  |  |  |  |  |  |  |  |  |  |  |  |  |
|  | Species | Specimen ID | Specimen source | Loci | | | | | | | | | | | | | | | | | | | | | | | | | | | |
|  |  |  |  | TOTAL NUMBER | ABCA1 | ADORA3 | AFF2 | APP | ATXN7 | AXIN1 | BCOR | CHRNA1 | DACH1 | DCTN2 | DENND5A | ERC2 | FAM123B | FBN1 | GHR | KCNMA1 | LRPPRC-171 | LUC7L | NPAS3.2 | PNOC | POLA1 | RAG2 | RPGRIP1 | SGMS1 | SIM1 | SMCX | ZIC3 |
| *OUTGROUP TAXA* | *Lemur catta* | LCT-10 | GenBank (Perelman et al., 2011) | 27 |  |  |  |  |  |  |  |  |  |  |  |  |  |  |  |  |  |  |  |  |  |  |  |  |  |  |  |
|  | *Propithecus verreauxi* | PVE-1 | GenBank (Perelman et al., 2011) | 27 |  |  |  |  |  |  |  |  |  |  |  |  |  |  |  |  |  |  |  |  |  |  |  |  |  |  |  |
|  | *Daubentonia madagascariensis* | DMD-5 | GenBank (Perelman et al., 2011) | 15 |  |  |  |  |  |  |  |  |  |  |  |  |  |  |  |  |  |  |  |  |  |  |  |  |  |  |  |
|  | *Macaca mulatta* | MMA-14 | GenBank (Perelman et al., 2011) | 27 |  |  |  |  |  |  |  |  |  |  |  |  |  |  |  |  |  |  |  |  |  |  |  |  |  |  |  |
|  | *Papio hamadryas* | PHM-1 | GenBank (Perelman et al., 2011) | 27 |  |  |  |  |  |  |  |  |  |  |  |  |  |  |  |  |  |  |  |  |  |  |  |  |  |  |  |
|  | *Theropithecus gelada* | TGE-2 | GenBank (Perelman et al., 2011) | 22 |  |  |  |  |  |  |  |  |  |  |  |  |  |  |  |  |  |  |  |  |  |  |  |  |  |  |  |
|  | *Chlorocebus aethiops* | CAE-4 | GenBank (Perelman et al., 2011) | 27 |  |  |  |  |  |  |  |  |  |  |  |  |  |  |  |  |  |  |  |  |  |  |  |  |  |  |  |
|  | *Homo sapiens* | HAS-34 | GenBank (Perelman et al., 2011) | 27 |  |  |  |  |  |  |  |  |  |  |  |  |  |  |  |  |  |  |  |  |  |  |  |  |  |  |  |
|  | *Pan troglodytes* | PTR-104 | GenBank (Perelman et al., 2011) | 27 |  |  |  |  |  |  |  |  |  |  |  |  |  |  |  |  |  |  |  |  |  |  |  |  |  |  |  |
|  | *Pongo pygmaeus* | PPY-155 | GenBank (Perelman et al., 2011) | 27 |  |  |  |  |  |  |  |  |  |  |  |  |  |  |  |  |  |  |  |  |  |  |  |  |  |  |  |
