# Supplementary material for: A multilocus phylogeny reveals deep lineages within African galagids (Primates: Galagidae)
Source: BMC Evol Biol. 2014 Apr 2;14:72. doi: 10.1186/1471-2148-14-72 (PMC4021292; doi:10.1186/1471-2148-14-72)
Supplement: Additional file 7 — List of the loci used in the coalescent-based species tree analyses for both datasets 27LOCI (Table S7a) and 19LOCI (Table S7b). For each dataset we report name of the locus, length (bp), number and percentage of constant, variable and parsimony informative characters. [file 1471-2148-14-72-S7.docx]

**Table S7a.** List of the 27 loci used in this study including total length, constant sites, variable sites and parsimony-informative sites.

| Locus | Taxon coverage | Length (bp) |  | Constant | |  | | Variable | |  | | Parsimony-informative | | |
| --- | --- | --- | --- | --- | --- | --- | --- | --- | --- | --- | --- | --- | --- | --- |
|  |  |  |  | bp | % |  | bp | | % |  | bp | | % |  |
|  |  |  |  |  |  |  |  | |  |  |  | |  |  |
| ABCA1 | 13 | 674 | 498 | 497 | 75.4 |  | 166 | | 24.6 |  | 89 | | 13.2 |  |
| ADORA3 | 15 | 416 | 441 | 347 | 83.4 |  | 69 | | 16.6 |  | 46 | | 11.1 |  |
| AFF2 | 16 | 510 | 799 | 441 | 86.5 |  | 69 | | 13.5 |  | 31 | | 6.1 |  |
| APP | 14 | 714 | 683 | 651 | 93.3 |  | 48 | | 6.7 |  | 19 | | 2.7 |  |
| ATXN7 | 15 | 565 | 292 | 474 | 84.2 |  | 89 | | 15.8 |  | 38 | | 6.7 |  |
| AXIN1 | 15 | 951 | 504 | 805 | 85.2 |  | 141 | | 14.8 |  | 63 | | 6.6 |  |
| BCOR | 15 | 789 | 499 | 683 | 86.6 |  | 106 | | 13.4 |  | 62 | | 7.9 |  |
| CHRNA1 | 16 | 425 | 525 | 294 | 73.9 |  | 111 | | 26.1 |  | 59 | | 13.9 |  |
| DACH1 | 15 | 630 | 642 | 501 | 79.7 |  | 128 | | 20.3 |  | 60 | | 9.5 |  |
| DCTN2 | 15 | 635 | 577 | 498 | 81.6 |  | 117 | | 18.4 |  | 72 | | 11.3 |  |
| DENND5A | 16 | 747 | 641 | 524 | 69.6 |  | 227 | | 30.4 |  | 110 | | 14.7 |  |
| ERC2 | 14 | 793 | 602 | 601 | 76.2 |  | 189 | | 23.8 |  | 105 | | 13.2 |  |
| FAM123B | 15 | 747 | 503 | 562 | 77.2 |  | 170 | | 22.8 |  | 104 | | 13.9 |  |
| FBN1 | 16 | 735 | 557 | 643 | 89.3 |  | 79 | | 10.7 |  | 42 | | 5.7 |  |
| GHR | 14 | 1295 | 678 | 1083 | 83.2 |  | 217 | | 16.8 |  | 106 | | 8.2 |  |
| KCNMA1 | 14 | 656 | 592 | 582 | 89.3 |  | 70 | | 10.7 |  | 45 | | 6.9 |  |
| LRPPRC-171 | 16 | 819 | 568 | 639 | 83.0 |  | 139 | | 17.0 |  | 65 | | 7.9 |  |
| LUC7L | 15 | 751 | 616 | 615 | 82.2 |  | 134 | | 17.8 |  | 78 | | 10.4 |  |
| NPAS3.2 | 16 | 680 | 273 | 498 | 74.1 |  | 176 | | 25.9 |  | 88 | | 12.9 |  |
| PNOC | 15 | 351 | 518 | 286 | 80.6 |  | 68 | | 19.4 |  | 44 | | 12.5 |  |
| POLA1 | 16 | 658 |  | 560 | 85.1 |  | 98 | | 14.9 |  | 58 | | 8.8 |  |
| RAG2 | 15 | 769 |  | 684 | 88.9 |  | 85 | | 11.1 |  | 45 | | 5.9 |  |
| RPGRIP1 | 15 | 713 |  | 591 | 83.5 |  | 118 | | 16.5 |  | 55 | | 7.7 |  |
| SGMS1 | 16 | 616 |  | 567 | 92.2 |  | 48 | | 7.8 |  | 24 | | 3.9 |  |
| SIM1 | 16 | 670 |  | 626 | 92.2 |  | 52 | | 7.8 |  | 19 | | 2.8 |  |
| SMCX | 16 | 365 |  | 273 | 80.0 |  | 73 | | 20.0 |  | 31 | | 8.5 |  |
| ZIC3 | 15 | 574 |  | 517 | 91.3 |  | 50 | | 8.7 |  | 19 | | 3.3 |  |
| TOTAL |  | 18248 | 15211 | |  |  | 3037 | |  |  | 1577 | |  |  |

**Table S7b** List of the 19 loci used in this study including total length, constant sites, variable sites and parsimony-informative sites.

| Locus | Length (bp) |  | Constant | |  | | Variable | | |  | | Parsimony-informative | | |
| --- | --- | --- | --- | --- | --- | --- | --- | --- | --- | --- | --- | --- | --- | --- |
|  |  |  | bp | % |  | bp | | % |  | | bp | | % |  |
|  |  |  |  |  |  |  | |  |  | |  | |  |  |
| AFF2 | 510 | 441 | 441 | 86.5 |  | 69 | | 13.5 |  | | 29 | | 5.7 |  |
| AXIN1 | 951 | 799 | 806 | 84.8 |  | 145 | | 15.2 |  | | 61 | | 6.4 |  |
| BCOR | 789 | 683 | 684 | 86.7 |  | 105 | | 13.3 |  | | 61 | | 7.7 |  |
| CHRNA1 | 425 | 292 | 313 | 73.6 |  | 112 | | 26.4 |  | | 57 | | 13.4 |  |
| DACH1 | 630 | 504 | 500 | 79.4 |  | 130 | | 20.6 |  | | 60 | | 9.5 |  |
| DCTN2 | 635 | 499 | 518 | 81.6 |  | 117 | | 18.4 |  | | 71 | | 11.2 |  |
| DENND5A | 747 | 525 | 525 | 70.3 |  | 222 | | 29.7 |  | | 107 | | 14.3 |  |
| FAM123B | 747 |  | 549 | 73.5 |  | 198 | | 26.5 |  | | 114 | | 15.3 |  |
| FBN1 | 735 | 642 | 658 | 89.5 |  | 77 | | 10.5 |  | | 40 | | 5.4 |  |
| KCNMA1 | 656 | 577 | 586 | 89.3 |  | 70 | | 10.7 |  | | 47 | | 7.2 |  |
| LRPPRC-171 | 819 | 641 | 680 | 83.0 |  | 139 | | 17.0 |  | | 65 | | 7.9 |  |
| LUC7L | 751 | 602 | 615 | 81.9 |  | 136 | | 18.1 |  | | 79 | | 10.5 |  |
| NPAS3.2 | 680 | 503 | 505 | 74.3 |  | 175 | | 25.7 |  | | 86 | | 12.6 |  |
| POLA1 | 658 | 557 | 560 | 85.1 |  | 98 | | 14.9 |  | | 57 | | 8.7 |  |
| RAG2 | 769 | 678 | 683 | 88.8 |  | 86 | | 11.2 |  | | 47 | | 6.1 |  |
| RPGRIP1 | 713 | 592 | 591 | 82.9 |  | 122 | | 17.1 |  | | 56 | | 7.9 |  |
| SGMS1 | 616 | 568 | 564 | 91.6 |  | 52 | | 8.4 |  | | 24 | | 3.9 |  |
| SIM1 | 670 | 616 | 620 | 92.5 |  | 50 | | 7.5 |  | | 18 | | 2.7 |  |
| ZIC3 | 574 | 273 | 525 | 91.5 |  | 49 | | 8.5 |  | | 20 | | 3.5 |  |
| TOTAL | 13075 |  | 10923 |  |  | 2152 | |  | 1099 | | | |  |  |
